# Integrated counter current separation and omics approaches for discovery of bioactive compounds from medicinal plants

**DOI:** 10.3389/fphar.2026.1901153

**Published:** 2026-09-28

**Authors:** Ananda Kumar Chettupalli, Priyanshu Pathak, Priya Sharma, Rajendra Kumar Jadi, Sunil Kumar Thota

**Affiliations:** 1 Department of Pharmaceutical Technology, School of Health and Medical Sciences, Adamas University, Kolkata, West Bengal, India; 2 Department of Pharmacy, School of Medical and Allied Health Sciences, Galgotias University, Greater Noida, Uttar Pradesh, India; 3 School of Pharmacy and Emerging Sciences, Baddi University of Emerging Sciences & Technology, Baddi, Himachal Pradesh, India; 4 Department of Pharmaceutics, School of Pharmacy, Anurag University, Hyderabad, Telangana, India; 5 Irma Lerma Rangel College of Pharmacy, Texas A&M Health Science Center, Texas A&M University, College Station, TX, United States

**Keywords:** AI, CCS, medicinal plant research, metabolomics-guided isolation, multi-omics technologies, natural product drug discovery, phytopharmaceutical development

## Abstract

Medicinal plants provide an important source of structurally varied bioactive metabolites with medicinal potential; however, their effective extraction, characterization, and standardization present considerable analytical challenges. Counter-current separation (CCS) methods, such as centrifugal partition chromatography (CPC) and high-speed counter-current chromatography (HSCCC), represent advanced liquid-liquid chromatographic approaches that minimize irreversible adsorption of samples. A systematic literature search was performed in PubMed/MEDLINE, Scopus, Web of Science Core Collection, ScienceDirect and Google Scholar using pre-defined search terms related to counter-current separation, six omics domains, artificial intelligence/machine learning, molecular networking, network pharmacology, regulatory systems, and industrial translation. We screened relevant studies using pre-defined eligibility criteria and thematically synthesized them according to methodological development, analytical integration, evidence maturity, regulatory relevance, and translational potential. These methods enable the effective purification of bioactive natural compounds, ensuring high recovery rates. Simultaneously, the development of metabolomics, transcriptomics, proteomics, genomics, lipidomics, and glycomics has significantly advanced the research on natural products, enabling detailed molecular profiling and systems-level biological interpretation. This review critically considers the use of CCS in combination with multi-omics technologies for the discovery of bioactive compounds from medicinal plants. Special attention is paid to solvent-system design, AI-aided process optimization, molecular networking, chemometric analysis, and bioactivity-guided fractionation strategies. In addition, the use of CCS in quality control, phytopharmaceutical standardization, and industrial-scale production is discussed. Integration of CCS with omics technologies, artificial intelligence (AI), and network-based analytical approaches offers a complementary platform for the enhancement of the discovery, prioritization, isolation, and characterization of bioactive natural products. These integrated workflows can facilitate candidate identification, structural characterization, mechanistic investigation and biological validation in early translational research. However, it should be kept in mind that the identification of a bioactive compound or fraction is not the same as drug discovery or therapeutic development. Further pharmacological, toxicological, pharmacokinetic, manufacturing, regulatory and clinical investigations are required for a purified natural product or botanical preparation to advance toward therapeutic development.

## Graphical Abstract

**Figure:**
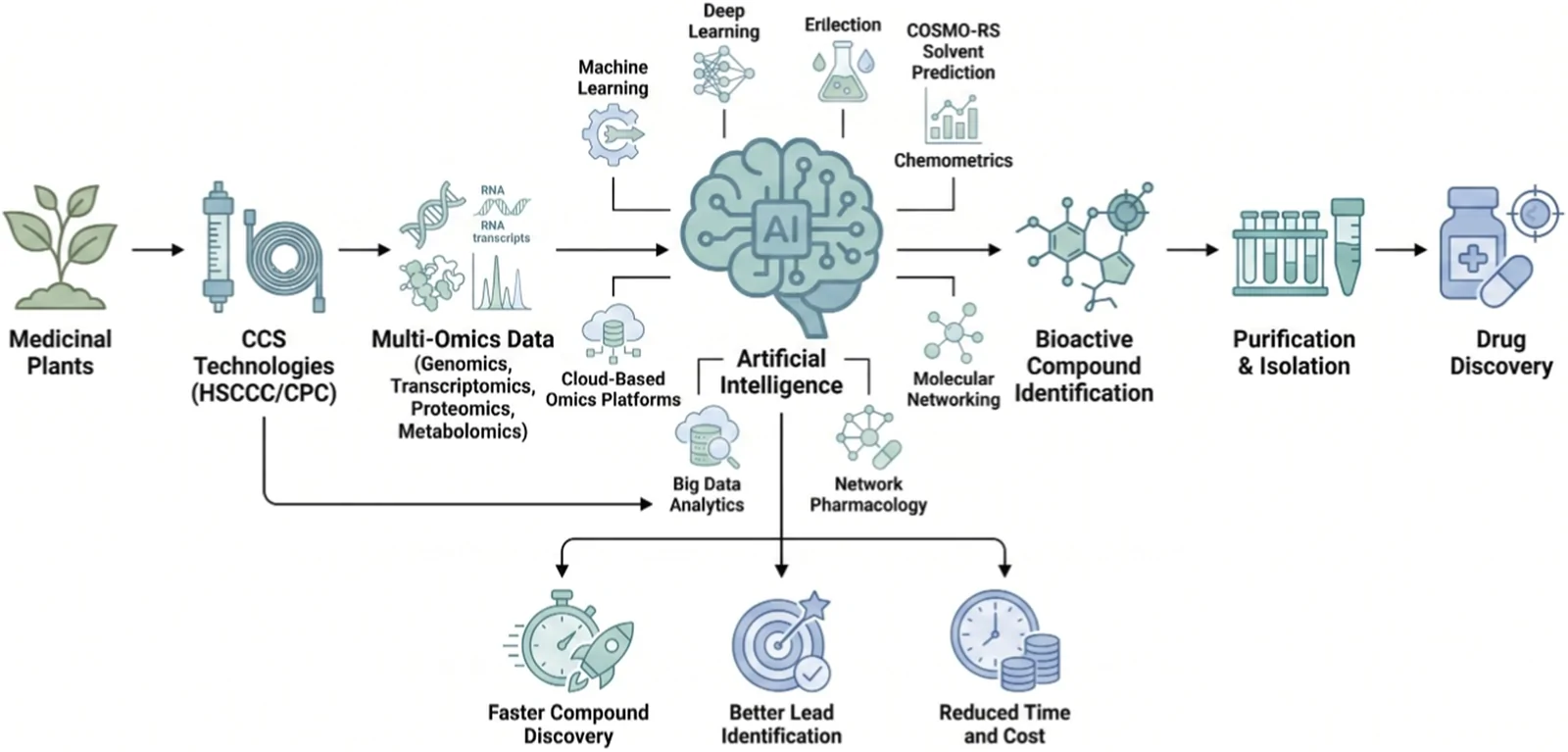

## 1 Introduction

Medicinal plants have been the main source of human therapeutics for thousands of years and evolutionary models for most present-day pharmacological drugs. Due to the metabolic plasticity of botanical organisms, they can synthesize an incredible variety of secondary metabolites, including complex alkaloids, flavonoids, terpenoids, and saponins. These phytochemicals have potent and diverse biological activities and applications in oncology, infectious diseases, and neurodegenerative disorders. Today, the process of identifying such bioactive molecules has evolved from serendipity and isolation-based phytochemistry to systems-level analyses (Luo et al., 2019). In addition to the increasing complexity of biological matrices, chemically diverse chemical constituents are needed for comprehensive profiling, targeted isolation, and structural elucidation to support modern drug discovery, which focuses on complex, multi-target disease paradigms (Wei et al., 2023).

Although chemicals in nature are greatly diverse, the extraction, separation and characterization of natural products are technically challenging and availability of large amounts of natural products is a serious bottleneck (Ning et al., 2018). Reverse-phase high-performance liquid chromatography (RP-HPLC) is one of the two most popular types of conventional liquid chromatography (LC) that employs a solid stationary phase. In traditional LC, the separation of analytes is based on interactions between the mobile and stationary phases, where the solid stationary phase is critical for LC (Li et al., 2022). Silica gel chromatography is widely used for the separation and purification of natural products due to its flexibility and high adsorption capacity (Vilas-Boas et al., 2022; Wang Y. et al., 2024). RP-HPLC has been used widely for phytochemical analysis and separation because of its better sensitivity, repeatability and resolution when compared to other methods (Wang et al., 2022a; Salem et al., 2020). Both of these methods suffer from some problems like irreversible adsorption of analytes on the solid stationary phase, limited sample loading capacity, and heavy consumption of solvents. These problems are particularly evident in the purification of natural compounds due to the often structurally complex and fragile nature of these compounds. Hence, techniques such as CCS that do not require supports for liquid separation have gained importance. One of the advantages of CCS is that it does not require a solid stationary phase and the liquid recovery and separation are better (Zhai et al., 2022). Solid supports possess chemisorptive and adsorptive properties which are very inconvenient for the research of natural products (Ning et al., 2018). Samples can be lost, structural degradation of labile metabolites can occur, recovery yields may be low, and chemical artifacts can be created during the separation process due to adsorption of the sample onto solid supports (Li et al., 2022). Research in functional foods, nutraceutical development, and drug discovery has been greatly propelled by the wide array of structurally varied phytochemicals and bioactive compounds found in plants from various geographical areas (Vilas-Boas et al., 2022).

Liquid-liquid partition chromatography (LPC), which is generally grouped under CCS, has become an innovative and highly scalable alternative to the physical and chemical constraints imposed by solid support matrices. HSCCC and CPC are techniques that use a biphasic liquid system in which one liquid phase is dynamically retained as the stationary phase by centrifugal force, and the other immiscible liquid phase is pumped through as the mobile phase (Wang Y. et al., 2024). CCS completely removes the matrix, providing improved sample recovery, high mass loadings, and minimal risk of compound denaturation and/or artifact formation. The absence of supports in this system enables the direct injection of crude extracts, significantly simplifying downstream processing.

Alongside the dramatic progress of separation science, the emergence and development of “omics” technologies, namely, metabolomics, proteomics, transcriptomics and genomics, have revolutionised the analytical depth with which medicinal plants can be explored (Wang et al., 2022a). Untargeted high-resolution mass spectrometry (HRMS) and nuclear magnetic resonance (NMR) metabolomics enable the production of a full “chemical map” of botanical extracts, which is the multidimensional space of the chemicals of an organism in a single analysis. Furthermore, genomics and transcriptomics provide deep genetic roadmaps that directly link plant taxonomy to chemical diversity and biological function by elucidating biosynthetic gene clusters that encode the enzymes involved in the enzymatic assembly of these metabolites (Salem et al., 2020).

The real paradigm shift in modern research on natural products is the orthogonal integration of highly scalable CCS platforms and advanced multi-omics workflows. Analytical metabolomics is invaluable for profiling complex mixtures but has limited preparative capabilities to generate sufficient quantities of target molecules for downstream pharmacological validation. In contrast, CCS is a very powerful purification method when applied without the need for a support. Still, it is greatly improved by the chemical intelligence it gains from prior mapping of the metabolome (Zhai et al., 2022). Hyphenated technologies, such as CCS with LC/MS, multidimensional gas chromatography, and systems-level network pharmacology, help researchers seamlessly transition between *in silico* annotation of bioactive features and direct, deterministic physical isolation, without the inefficiencies of blind fractionation.

This review article critically examines the methodological synergies between CCS technologies and advanced multi-omics platforms in medicinal plant research. The report explains the basic thermodynamic and hydrodynamic principles of liquid-liquid partitioning, summarizes recent highly advanced developments in machine learning (ML)-based solvent system selection, and discusses the in-depth integration of metabolomics, molecular networking, and systems biology. Overall, the integration of all these analytical areas offers a powerful, reproducible, and highly scalable tool for the fast discovery, isolation, and pharmacologic standardization of bioactive compounds from medicinal plants.

## 2 Literature search strategy and study selection

### 2.1 Review objective and scope

This review was undertaken to comprehensively examine the development and integration of counter-current separation (CCS) technologies with multi-omics approaches, artificial intelligence (AI) and machine learning (ML), molecular networking, network pharmacology, regulatory frameworks, and industrial translation in natural-product and botanical-drug research. The literature search was designed to capture evidence concerning CCS methodologies, including high-speed counter-current chromatography (HSCCC), centrifugal partition chromatography (CPC), pH-zone-refining counter-current chromatography, and related liquid–liquid separation approaches, as well as their integration with genomics, transcriptomics, proteomics, metabolomics, lipidomics, and glycomics. Additional searches addressed AI/ML-assisted CCS optimization, molecular networking, systems biology, network pharmacology, quality control, regulatory considerations, scale-up, and clinical or industrial translation.

### 2.2 Literature databases and search strategy

A systematic literature search was performed in PubMed/MEDLINE, Scopus, Web of Science Core Collection, ScienceDirect and Google Scholar to identify relevant publications on CCS, omics-integrated natural-product research, AI/ML assisted separation, molecular networking, network pharmacology, regulatory considerations and industrial translation. The literature search was limited to the period between 2010 and 2026. We also screened the reference lists of relevant review articles and primary studies for potentially relevant publications that were not identified in the electronic database searches (Table 1).

**TABLE 1:** Eligibility criteria used for literature selection.

Criterion	Inclusion	Exclusion
Publication type	Original research, systematic reviews, narrative reviews, methodological papers, regulatory/guideline documents	Editorials, letters, conference abstracts without sufficient data
Topic	CCS, HSCCC, CPC, CCS–omics, AI/ML, molecular networking, network pharmacology, regulation, quality control, industrial translation	Studies unrelated to the review scope
Biological source	Medicinal plants, botanical extracts, natural products, marine/fungal natural products where relevant	Purely synthetic compounds without relevance to natural-product separation
Omics	Genomics, transcriptomics, proteomics, metabolomics, lipidomics, glycomics, multi-omics	Unrelated high-throughput technologies
Analytical approaches	CCS coupled with MS, NMR, omics, chemometrics, molecular networking or computational approaches	Studies without relevance to the review objectives
Regulatory evidence	FDA, EMA, ConPhyMP and relevant quality/GMP frameworks	Non-authoritative regulatory claims without supporting documentation
Language	English	Non-English articles where reliable translation/full-text assessment was unavailable
Date	[2010–2026]	Outside the predefined period unless considered a seminal/key reference

### 2.3 Search terms and Boolean operators

#### 2.3.1 Search block 1: CCS

(“counter-current chromatography” OR “countercurrent chromatography” OR “counter-current separation” OR “countercurrent separation” OR HSCCC OR CPC OR “centrifugal partition chromatography” OR “high-speed counter-current chromatography” OR “pH-zone-refining counter-current chromatography”) AND (“natural product*” OR phytochemical* OR medicinal plant* OR botanical* OR phytopharmaceutical*).

#### 2.3.2 Search block 2: CCS + omics

(“counter-current chromatography” OR HSCCC OR CPC OR “centrifugal partition chromatography”) AND (omics OR metabolomic* OR genomic* OR transcriptomic* OR proteomic* OR lipidomic* OR glycomic* OR “multi-omics” OR “multiomics”).

#### 2.3.3 Search block 3: CCS + AI/ML

(“counter-current chromatography” OR HSCCC OR CPC) AND (“artificial intelligence” OR “machine learning” OR “deep learning” OR chemometric* OR “predictive model*” OR “digital twin*” OR “reinforcement learning”).

#### 2.3.4 Search block 4: molecular networking

(“counter-current chromatography” OR HSCCC OR CPC) AND (“molecular networking” OR “global natural products social molecular networking” OR GNPS OR “bioactive molecular networking”).

#### 2.3.5 Search block 5: network pharmacology

(“counter-current chromatography” OR HSCCC OR CPC OR “natural product*” OR phytochemical*) AND (“network pharmacology” OR “systems pharmacology” OR “systems biology” OR “compound-target-pathway”).

#### 2.3.6 Search block 6: regulation and quality

(“botanical drug*” OR “botanical medicine*” OR “herbal medicinal product*” OR phytopharmaceutical* OR “medicinal plant extract*”) AND (regulation OR regulatory OR GMP OR “quality control” OR standardization OR ConPhyMP OR FDA OR EMA).

#### 2.3.7 Search block 7: industrial translation

(“counter-current chromatography” OR HSCCC OR CPC) AND (“scale-up” OR industrial* OR manufacturing OR GMP OR commercialization OR “process development” OR “continuous processing” OR translation OR “drug development”).

### 2.4 Screening and study selection

All retrieved records were screened following a predefined selection process. Duplicate records identified across databases were removed before title and abstract screening. Titles and abstracts were initially screened for relevance to the review objectives, followed by full-text assessment of potentially eligible publications. Studies were excluded when they did not address CCS, omics-integrated natural-product research, AI/ML, molecular networking, network pharmacology, regulatory considerations, quality control, or industrial translation within the defined scope. Reference lists of key reviews and highly relevant primary studies were also examined manually to identify additional publications. The final literature set was organized according to the major thematic domains of the review (Natukunda and Muchene, 2023).

#### 2.4.1 Definition and classification of genuine CCS–omics integration

To specifically assess the extent of genuine integration between counter-current separation (CCS) and omics technologies, eligible publications were further classified according to the depth of methodological integration. A study was categorized as demonstrating genuine CCS–omics integration when CCS, including HSCCC or CPC, formed an explicit component of the experimental workflow and the resulting fractions or separated materials were subsequently subjected to an omics-level analytical platform, with the generated data used for systematic molecular profiling, compound annotation, pathway interpretation, biomarker discovery, or biological/mechanistic characterization. Studies in which CCS was used solely for preparative purification followed by conventional MS, NMR, or targeted identification of individual compounds were classified separately as CCS-assisted chemical profiling rather than genuine CCS–omics integration. Conventional CCS studies focused primarily on isolation and structural characterization were classified as CCS purification/isolation studies (Table 2) (Fu et al., 2026).

**TABLE 2:** Classification of published studies according to the degree of CCS–omics integration.

Classification	Definition	Typical workflow	Interpretation	References
Genuine CCS–omics integration	CCS fractions directly subjected to an omics platform with systematic data interpretation	Extract → HSCCC/CPC → untargeted metabolomics → annotation/pathway analysis	True CCS–omics workflow	Leontyev et al. (2025)
CCS-assisted molecular profiling	CCS followed by broad MS/NMR profiling but without a complete omics framework	Extract → HSCCC/CPC → MS/MS + NMR → dereplication	Integrated chemical profiling	Kartowikromo et al. (2023)
CCS-assisted targeted analysis	CCS followed by identification/quantification of selected compounds	Extract → CCS → HPLC-MS/NMR	Not considered omics	Kartowikromo et al. (2023)
Conventional CCS purification	CCS primarily used for isolation/purification	Extract → HSCCC/CPC → purified compound → structure elucidation	Not CCS–omics	Friesen et al. (2015)

This classification was introduced because the terms “metabolomics,” “MS profiling,” and “chemical profiling” are not interchangeable. In particular, the use of MS or NMR after CCS does not automatically establish an omics workflow. The classification therefore emphasizes the extent to which CCS and omics datasets were functionally integrated within the same analytical or biological workflow.

### 2.5 Data extraction and thematic synthesis

Data were obtained from qualifying publications in alignment with the aims and thematic framework of the review. The gathered data encompassed the year of publication, source of natural products, characteristics of the CCS technique and solvent system when available, target compound or class of phytochemicals, analytical platform, omics domain, AI/ML methodology, molecular networking or network pharmacology strategy, key analytical or biological results, noted limitations, regulatory significance, and implications for industry or translation. The gathered evidence was systematically categorized into CCS technique, CCS–omics integration, AI/ML applications, molecular networking, network pharmacology, bioactivity-guided discovery, quality assurance, regulatory factors, industrial scaling, and clinical application (Figure 1) (Adam and Ozarisoy, 2023).

**FIGURE 1:**
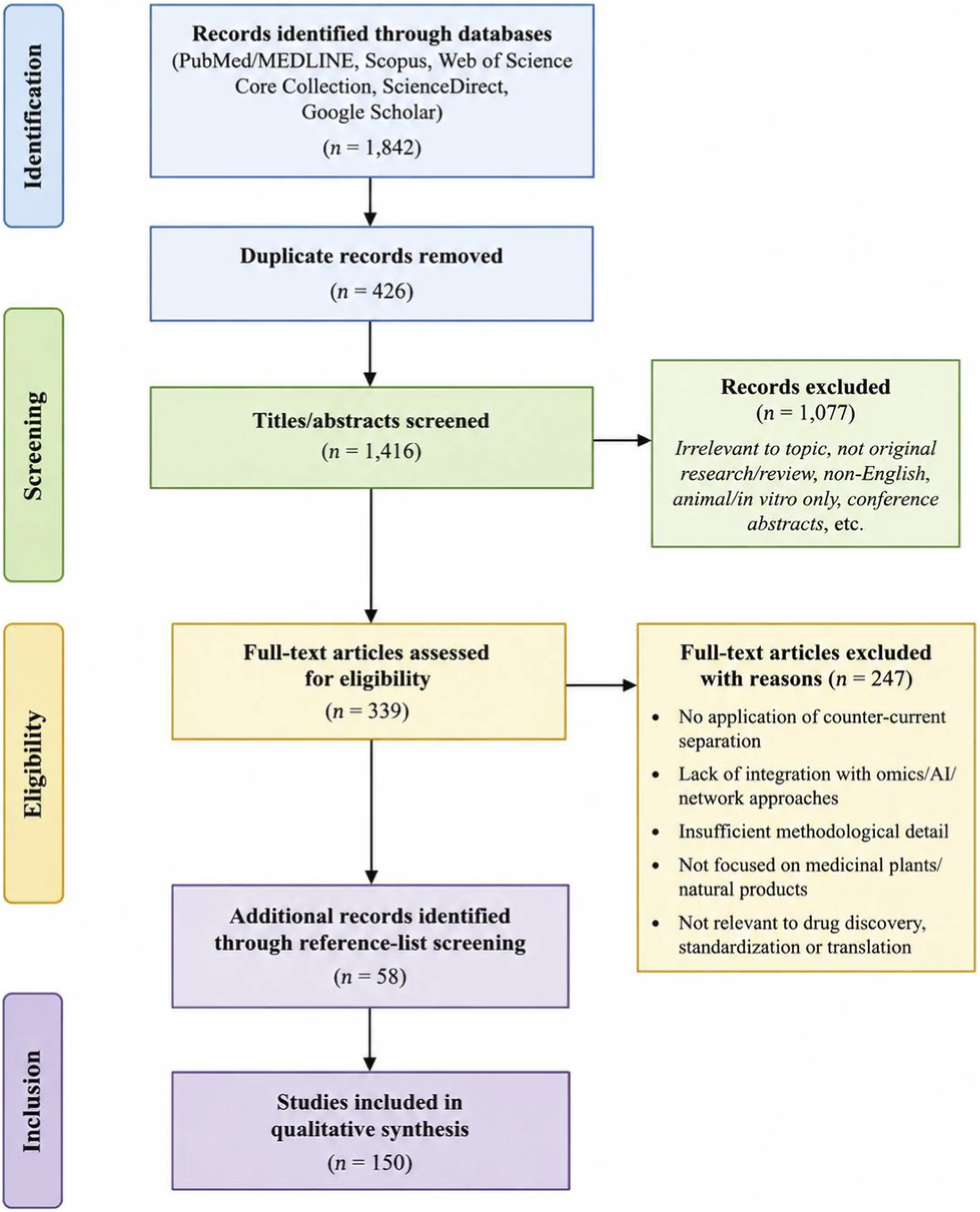
PRISMA-style flow diagram of the literature identification, screening, eligibility assessment, and inclusion process.

### 2.6 Quality assessment and evidence classification

Because this review encompasses diverse evidence types, including methodological studies, analytical workflows, omics studies, computational investigations, regulatory documents, and industrial reports, a single risk-of-bias instrument was not considered applicable to all evidence categories. Instead, the strength and maturity of evidence were qualitatively classified according to study design, experimental validation, independent validation, reproducibility, and translational relevance (Table 3). Established applications supported by experimental validation were distinguished from emerging proof-of-concept approaches, computational predictions, and future-oriented concepts. Particular attention was given to independent validation and reproducibility when interpreting AI/ML-based approaches and omics-derived biomarkers (Ma et al., 2020).

**TABLE 3:** Literature-search domains, representative search terms, and purpose.

Search domain	Main search concepts	Purpose	References
CCS	CCS, HSCCC, CPC, counter-current chromatography	Identify separation technologies	Friesen et al. (2015)
CCS–omics	CCS + metabolomics/genomics/transcriptomics/proteomics/lipidomics/glycomics	Identify integrated omics applications	Dai and Shen (2022)
AI/ML	CCS + AI/ML/deep learning/chemometrics	Identify computational applications	Houhou and Bocklitz (2021)
Molecular networking	CCS + GNPS/molecular networking/BMN	Identify dereplication and bioactivity-networking studies	Gaudêncio et al. (2023)
Network pharmacology	CCS/natural products + network pharmacology/systems biology	Identify mechanism-oriented studies	Panossian (2026)
Regulation	Botanical drugs + FDA/EMA/ConPhyMP/GMP	Identify regulatory and quality requirements	Center for Drug Evaluation and Research (2020)
Industrial translation	CCS + scale-up/manufacturing/GMP/continuous processing	Identify translational and manufacturing evidence	Ghavami et al. (2026)

## 3 Fundamentals of CCS technologies

### 3.1 Terminology and classification of counter-current separation technologies

The terminology associated with liquid-liquid separation technologies is often inconsistently used in the literature. Researchers use inconsistent terminology when referring to the technologies of liquid–liquid separation. Inconsistently in the literature. The literature often inconsistently uses the terminology associated with liquid–liquid separation technologies. Inconsistently in the literature. Therefore, clear definitions are necessary to distinguish related but non-identical concepts.

Counter-current separation (CCS) is the broadest term and refers to separation techniques that employ two immiscible liquid phases without a solid stationary phase. Solute separation occurs through repeated partitioning between the mobile and stationary liquid phases.

Counter-current chromatography (CCC) is a subclass of CCS in which the stationary liquid phase is retained through hydrodynamic mechanisms generated by planetary motion. High-speed counter-current chromatography (HSCCC) is the classical implementation of this principle, whereas high-performance counter-current chromatography (HPCCC) represents an advanced form operating at higher rotational speeds and enhanced centrifugal fields, resulting in improved stationary-phase retention and separation efficiency (Debnath et al., 2025).

Centrifugal partition chromatography (CPC) differs mechanistically from CCC because stationary-phase retention is achieved through hydrostatic centrifugal forces in a series of interconnected chambers rather than through hydrodynamic equilibrium in coiled tubing.

The term “liquid–liquid partition chromatography” describes the fundamental separation principle common to these techniques, namely, solute partitioning between two immiscible liquid phases. However, it should not be considered synonymous with CCS because other liquid–liquid separation methods may also rely on partition phenomena.

Throughout this review, the term “CCS” is used when discussing the field collectively, whereas “HSCCC,” “HPCCC,” and “CPC” are employed when referring to specific instrumental platforms (Hammerschick and Vetter, 2022).

### 3.2 Principles of liquid-liquid partition chromatography

The principle of counter-current chromatography (CCC) is that of solute partitioning between two immiscible liquid phases and is applied solely to this thermodynamic principle. The partition coefficient (*K or K*_*D*_) is the basic parameter that determines this mass transfer process and is defined as the ratio of the concentration of the solute in the stationary phase (*c*_*s*_) to the concentration of the solute in the mobile phase (c_m_), which can be expressed mathematically as *K = c*_*s*_/*c*_*m*_. The specific K value of the analyte essentially determines the flow rate of the analyte through the column (Acevedo, 2026). The higher the *K*, the more slowly the analytes are eluted from the column as they are more attracted to the stationary phase, and the lower the *K*, the more rapidly the analytes are eluted from the column as they are more attracted to the mobile phase. The optimization of this coefficient to the “sweet spot” is the basic principle of CCS methodology, which is usually set within the range of −0.4 ≤ log_10_ K_p_ ≤ +0.4 (Choi et al., 2024).

### 3.3 Historical development of CCS techniques

Dr. Yoichiro Ito laid the conceptual and engineering basis for modern CCS through his pioneering research, which led to the invention of the flow-through planetary centrifuge. Ito noted that when the coiled tube is placed in a centrifugal field generated by a planet, the complex epicyclic motion of the tube leads to the clear separation of the light and heavy liquid phases and the development of unique hydrodynamic phenomena at the “head” and “tail” of the tube (The Analytical Scientist, 2026). This was an essential innovation that addressed early problems of low flow rate and poor mixing in droplet counter-current chromatography (DCCC). By combining stationary inflow and outflow lines, and a rotating coil in a planetary motion, the flow-through coil planetary centrifuge was born, ushering in the modern age of HSCCC (Friesen et al., 2015).

### 3.4 High-speed CCC

HSCCC is a type of CCS instrument and is hydrodynamic. They use a force achieved by the rotation of an Archimedes’ screw within a continuous helical coil, which rotates on two axes at the same time, in the true planetary motion. This complex epicyclic motion generates highly variable centrifugal forces that continuously drive active mixing and settling of the two liquid phases, yielding up to 4 theoretical plates/tube turn. Although early HSCCC machines weighed around 80 g, modern HPCCC machines can achieve forces of 240 g. This huge gain in g-force, along with larger column bores, allows for tenfold higher throughput, significantly higher mobile-phase flow rates, and superior stationary-phase retention (Yao et al., 2012). Because of the constantly recurring mechanical stresses of planetary rotation, the connecting tubing must be replaced periodically at low pressures (0.1–10 kg/cm^2^) at which HSCCC operates.

### 3.5 CPC

Unlike the multi-axis hydrodynamic of HSCCC, CPC is a hydrostatic method. CPC operates on one axis of rotation, which creates a constant, even and very stable centrifugal force field. The CPC rotor is not a single helical coil, but a set of connected discrete extraction chambers (or cells) connected by thin ducts (Table 4). The stationary phase is immobilized in these discrete cells by strong centrifugal forces, and the mobile phase is pumped through the cells, bubbling or cascading through the stationary phase, allowing for the mass transfer to occur. CPC units can be operated at higher operating pressures (2–70 kg/cm^2^), provide extremely steady stationary-phase retention, and can reach up to 1 theoretical plate per channel (Kostanyan et al., 2020).

**TABLE 4:** Comparative operational and structural characteristics of hydrodynamic HSCCC and hydrostatic CPC systems used in liquid-liquid separation of natural products.

Parameter	HSCCC (hydrodynamic)	CPC (hydrostatic)	References
Axis of rotation	Two axes (epicyclic planetary motion)	One axis (simple rotation)	Kiene et al. (2023)
Centrifugal field	Variable	Constant, stable	Hammerschick and Vetter (2022)
Column geometry	Continuous helical tubing	Discrete, interconnected chambers	Kiene et al. (2023)
Efficiency	Up to 4 plates per tube turn	Up to 1 plate per channel	Kiene et al. (2023)
Operating pressure	Low (0.1–10 kg/cm^2^)	Medium to High (2–70 kg/cm^2^)	​
Stationary phase retention	Variable	Good	Hammerschick and Vetter (2022)
Maintenance profile	Connecting tubing requires periodic replacement	Rotating rotary seals require periodic lubrication	Kiene et al. (2023)

### 3.6 Advantages of CCS over conventional chromatography

The major difference among all CCS techniques is that they do not require a solid support matrix. The physical characteristic means that irreversible sample adsorption is absent, thus making CCS a high-recovery, totally non-destructive preparative method that is extremely suitable for the isolation of labile or highly reactive phytochemicals (Table 5). In addition, the capacity of CCS is extraordinary, as the entire internal volume can serve as the active stationary phase, allowing crude botanical extracts to be injected in large amounts without significant sample loss, thereby reducing processing time because there is no need for deep prepurification (Mondal et al., 2026).

**TABLE 5:** Comparative advantages of CCS techniques, including HSCCC and CPC, over conventional chromatographic methods for phytochemical purification and natural product isolation.

Parameter	HSCCC	CPC	Preparative HPLC	Silica gel chromatography	References
Stationary phase	Liquid	Liquid	Solid	Solid	Li et al. (2021)
Sample recovery	Very high	Very high	Moderate	Moderate	Thiébaut and Rosset (1992)
Irreversible adsorption	None	None	Possible	High	Manousi et al. (2022)
Processing of crude extracts	Excellent	Excellent	Limited	Moderate	Zhang et al. (2023)
Scale-up capability	High	Very high	Moderate	Low	He et al. (2024)
Solvent consumption	Moderate	Moderate	High	High	Lefebvre et al. (2021)
Throughput	High	High	Moderate	Low	Lefebvre et al. (2021)
Suitable for labile compounds	Excellent	Excellent	Moderate	Poor	McAlpine et al. (2012)

CCS has many advantages over solid-phase chromatography, including less irreversible adsorption, better sample recovery, and preservation of chemically unstable metabolites; however, it is not a one-size-fits-all solution for preparative HPLC and other chromatographic methods. The strengths and drawbacks of each separation strategy depend on the physicochemical properties of the analytes, sample complexity, required separation resolution, throughput, and applications. Due to its resolution, automation, and reproducibility, preparative HPLC is still used in many analytical and medicinal applications. CCS preparative purification works best for irreversible adsorbable natural compounds. Instead of competing methodologies, these should be complementary, with the analytical goal determining which one is used. CCS has shown promise as a replacement for solid-phase chromatography due to its enhanced sample recovery and absence of irreversible adsorption; however, it is still not extensively employed because of several problems. Optimization of biphasic solvent systems is still a time-consuming, empirical, and difficult procedure for method development. CCS performs poorly compared to preparative HPLC for molecules with similar partition coefficients, and long separation durations can limit sample throughput. Instrumentation is expensive and requires experienced personnel to produce reliable results. Without certified solvent system databases and conventional operating processes, normal industrial applications are confronted with obstacles. Future research should increase CCS reproducibility, scalability, and industry acceptance by AI-assisted solvent selection, automated process optimization, and equipment design.

### 3.7 Solvent system theory and partition coefficients

The key to the success of any CCS separation is essentially the choice of an optimal biphasic solvent system that results in the desired range of partition values for the target compounds. The thermodynamics of solute partition is not the only thing that is determined by the solvent system, but also the physical hydrodynamics of the column. The capillary wavelength and the volume of the stationary phase retained during mobile-phase elution are directly affected by critical physical parameters such as interfacial tension (γ), the density difference between the upper and lower phases (Δρ), and the settling time of the emulsion (Hopmann et al., 2010). Settling time has traditionally been used to estimate stationary-phase retention; however, work on high-resolution HSCCC separations shows that settling is not always inversely proportional to actual retention, suggesting complex flow phenomena under high centrifugal forces.

## 4 Methodological advances in CCS

### 4.1 Solvent system selection strategies

Traditionally, the most important problem in the operation of CCS has been the empirical search for the optimum biphasic system. For this purpose, standardized solvent families are developed to cover a broad and predictable range of polarities. HEMWat is a continuous polarity gradient from highly non-polar (HEMWat 5) through intermediate polarities (HEMWat 0) to highly polar mixtures (HEMWat +7) (López-López et al., 2020). Likewise, the liquid system used in Arizona is universal and includes 23 different volumetric compositions (A–Z) of heptane, ethyl acetate, methanol, and water. The basic thermodynamic principle for the Arizona system is that the moles of methanol to water are equal to the moles of heptane to ethyl acetate (Table 6) (Friesen et al., 2015). This relationship ensures that the lower-phase composition is also consistent, meaning that the lower-phase density characteristics are the same across all compositions, irrespective of the alkane used.

**TABLE 6:** Common biphasic solvent systems employed in CCS, their polarity ranges, major phytochemical applications, and suitability for natural product purification.

Solvent system	Composition	Polarity range	Major applications	References
HEMWat	Hexane-ethyl acetate-methanol-water	Wide	General phytochemical separation	Han et al. (2023)
Arizona system	Heptane-ethyl acetate-methanol-water	Wide	Standardized CCS screening	Wang et al. (2020)
Chloroform-methanol-water	Variable ratios	Medium	Alkaloids	Vilas-Boas et al. (2022)
TBME-BuOH-CAN-water	1:2:1:5	High	Saponins and glycosides	Chibério et al. (2020)
Hexane-ethyl acetate-methanol-water	Variable ratios	Low to medium	Terpenoids and flavonoids	Kiene et al. (2023)
DES-based systems	DES-water-organic solvent	Variable	Green extraction and purification	Toribio et al. (2021)

### 4.2 Quantitative performance comparison of CCS with conventional chromatographic techniques

CCS uses liquid-liquid partitioning instead of solid-phase chromatography to avoid analytes becoming irreversibly bonded to stationary phases. Compared with conventional solid-phase chromatographic techniques, countercurrent separation (CCS) generally provides higher sample recovery because separation occurs in the absence of a solid stationary phase, thereby minimizing irreversible adsorption and sample loss. Multiple experimental studies have demonstrated that CCS is capable of achieving near-quantitative recovery for many classes of natural products, although recovery varies depending on solvent system selection, physicochemical properties of the analytes, and operating conditions. This enhancement is essential for low-abundance, thermolabile, and highly polar compounds that are irreversibly adsorbable during standard purification (Table 7) (Friesen et al., 2015).

**TABLE 7:** Quantitative comparison of CCS and conventional chromatographic techniques based on representative literature reports.

Performance parameter	CCS (HSCCC/CPC)	Silica gel chromatography	Preparative HPLC	Representative references
Sample recovery (%)	95%–99%	70%–90%	85%–98%	Berthod and Hassoun (2006)
Product purity (%)	90%–99%	80%–95%	95%–99%	Berthod et al. (2007)
Irreversible adsorption	None	Moderate-High	Low	Maryutina and Rakcheev (2005)
Number of purification steps	1–2	2–5	1–3	Liu Y. et al. (2015)
Sample loading capacity	High (g-kg scale)	Moderate	Moderate	Berthod (2007)
Solvent system optimization	Required	Moderate	Moderate	Ito (2005)
Stationary phase replacement	Not required	Required	Required	Alain Berthod (2011)

CCS can provide highly pure fractions with a purity of 90% to over 98% after one separation step, reducing the need for several chromatographic purifications, according to several comparative studies. Although silica gel chromatography can isolate structurally identical phytochemicals, several purification steps are frequently needed to reach equal purity. CCS reduces solvent usage, processing time, and sample handling while improving purification efficiency. Recent advancements in loadability and scale-up have considerably increased analytical throughput in CPC. Preparative CPC systems have great recovery and repeatability for grams to kilograms samples. CCS has a higher loading capacity and lower operational costs for large-scale natural product isolation because there is no solid stationary phase to replenish or regenerate, but preparative HPLC has better chromatographic resolution for closely related chemicals. However, traditional chromatography may not be better than CCS. Separation efficiency depends on choosing a biphasic solvent solution, which may require significant experimental screening. Additional purification of substances with similar partition coefficients may require preparative HPLC (Skalicka-Woźniak and Garrard, 2014). CCS should be used in conjunction with other separation tactics, not in instead of them. Sample complexity, purification goals, and downstream analytical needs determine the method.

### 4.3 Critical interpretation of quantitative performance

The quantitative comparison indicates that CCS offers a major advantage in sample recovery and preservation of labile phytochemicals because the separation process does not depend on a solid stationary phase. The representative recovery range reported for CCS (95%–99%) is higher than that reported for silica-gel chromatography (70%–90%) and is comparable with preparative HPLC (85%–98%). However, the advantage of CCS is not universal. Preparative HPLC may provide superior resolution for closely related compounds with similar physicochemical properties, whereas CCS may require extensive biphasic solvent-system optimization. Thus, CCS should be seen as a complementary rather than a universally superior separation platform. Choice of CCS, preparative HPLC, or silica‐based chromatography should depend on compound polarity, partition coefficient, sample complexity, required purity, loading capacity, throughput, and downstream analytical requirements (Huang et al., 2022).

### 4.4 Green and sustainable solvent systems

With the global sustainability movement and the principles of green chemistry, modern CCS workflows are increasingly replacing volatile halogenated organic compounds with more environmentally friendly alternatives. Deep Eutectic Solvents (DES) are mixtures of organic compounds that exhibit melting points lower than expected for their constituent parts, and are highly effective extraction media. The most significant obstacle to DES use was the high viscosity of the DES itself, which made it difficult to separate target solutes from the DES matrix (Hertel et al., 2012). But in the case of HSCCC, DES can be integrated directly, enabling simultaneous recovery of extracted compounds and recycling of the DES. For instance, using a solvent system specifically created for the purpose that contains choline chloride/L-malic acid, water and ethyl acetate in a green manner, a DES extract of chrysanthemum was successfully separated, and both the active phytochemicals and the original DES matrix were recovered. Also, DES made from choline chloride and 1,4-butanediol was successfully applied in combination with the quaternary solvent system to extract flavonoids from the thorns of Gleditsia sinensis (Yu et al., 2021a).

### 4.5 pH-zone refining and gradient elution

The standard CCS is governed by linear partitioning isotherms, yielding standard Gaussian-shaped peaks as expected. The pH-zone refining technique is a specialized mode of ion-exchange displacement chromatography that dramatically increases mass-loading capacity, particularly for the displacement of ionizable compounds (such as alkaloids or organic acids) (Ito and Ma, 1996). This method is based on the ionic modification of the stationary phase (using an “ionic retainer” such as trifluoroacetic acid, di(2-ethylhexyl) phosphoric acid, etc.) and the mobile phase (using an “ionic eluter” or “displacer” such as triethylamine, ammonia, etc.). The mobile phase continues to move through the column and forms sharp dynamic pH fronts (an isotachic train), which force the ionizable analytes to elute as a series of highly concentrated, contiguous rectangular blocks governed exclusively by their pK_a_ and hydrophobicity. This technique can boost sample loading by 20-fold or more compared with traditional HSCCC and often concentrates trace impurities at the interfaces between the main rectangular peaks (Friesen et al., 2015).

### 4.6 Process optimization and automation

Advanced multi-mode separation techniques are increasingly being used to maximize throughput and efficiency. However, in an overlapping elution-extrusion counter-current chromatography (EECCC), the standard elution is continued up to a predetermined switch volume after which the remaining stationary phase is “extruded” from the column by pumping the organic phase at a high flow rate, thus recovering highly retained lipophilic solutes in a short time without consuming excessive volumes of organic solvents. This method provides the effect of an infinite separation window, enabling recovery of compounds with an infinite partition coefficient within a very concentrated band. Other automated modes are dual-mode and flow reversal, in which the fluid flow is periodically reversed and the roles of the mobile and stationary phases are alternated to extract polar and non-polar constituents from the same injection sequentially (Sun et al., 2020).

### 4.7 Scale-up and industrial applications

There are a few surprises when scaling up from a benchtop liquid-liquid model to an industrial preparative or pilot-scale setup. The basic thermodynamics of the liquid phases (in contrast to the fluid dynamics of channelling and extreme pressure drops for solid-packed beds) are completely independent of the column volume, allowing scale-up to be a linear mathematical exercise. The separation resolution remains the same when changing from an analytical 10 mL coil to a multi-liter pilot CPC rotor by making the flow rates and injection volumes proportional (Kaśkosz et al., 2023). This continuous predictive modelling enables continuous processing methods, such as twin-column continuous chromatography and recycling CCC, to be used for the industrial-scale purification of high-value bioactive natural products and pharmaceutical intermediates (Bržezická et al., 2023).

### 4.8 AI-assisted and computational optimization of CCS

The commonly used empirical “shake flasks” method of screening biphasic mixtures is extremely time-consuming and solvent-intensive. One of the key developments in predictive separation science has been the combination of the Conductivity-like Screening Model for Real Solvents (COSMO-RS). It is a powerful computational framework that employs state-of-the-art quantum chemistry and statistical thermodynamics to calculate the chemical potential, activity coefficients and free energy of solvation (ΔΔG^‡^_solv_) of solutes in complex biphasic mixtures, directly from the 3D structure of the molecule. COSMO-RS drastically reduces the experimental design space by computationally simulating the entire matrix of the Arizona or HEMWat *in silico*. Further elaborations, such as COSMOmic, can model anisotropic phases and accurately predict partition coefficients across complex lipid bilayers and micellar structures (Win et al., 2023; Müller et al., 2025).

Optimization is indeed solved using AI and ML, and the applications extend beyond physics and thermodynamics. A deep learning architecture based on 1D molecular representations, such as Extended Connectivity Fingerprints (ECFP), and the Long Short-Term Memory (LSTM) network has enabled the successful prediction of partition coefficients with improved accuracy (Tawalbeh et al., 2025). Moreover, Bayesian Experimental Design (BED) models trained with Gaussian Processes (GP) explore thousands of theoretical solvent configurations, predict equilibrium states and partition coefficients, and use only 1% of the designed solvent space for sampling.

AI can improve chromatography and predictive modelling and may improve the efficiency of natural product discovery. However, the performance of the model depends greatly on the quality, diversity and the number of datasets used for training. AI models are frequently black-box systems, having low interpretability which makes it impossible to explain prediction outputs, eroding regulatory and therapeutic trust. Issues include heterogeneity of data, algorithmic bias, overfitting, absence of external validation and paucity of high-quality annotated datasets. There are no reporting standards or repeatable operations available for applying AI to pharmaceutical research. Future research should focus on enhancing the reliability and applicability of the presented work, explainable AI, standardized datasets, well-defined validation methodologies and experimental evidence integration (Mullowney et al., 2023).

### 4.9 Omics technologies in medicinal plant research

#### 4.9.1 Metabolomics for natural product profiling

Metabolomics has become one of the most powerful omics technologies for the comprehensive characterization of medicinal plants. It is specific to the analysis of all low-molecular-weight metabolites in the biological system and offers a direct representation of the plant’s biochemical status (Wang et al., 2022a). A new generation of highly advanced analytical platforms, such as ultra-HPLC coupled with quadrupole time-of-flight mass spectrometry (UHPLC-QTOF-MS), gas chromatography-mass spectrometry (GC-MS) and NMR spectroscopy, enables the simultaneous detection of thousands of metabolites - alkaloids, flavonoids, terpenoids, phenolic acids and glycosides. Metabolomic profiling has proven to be of great value in identifying bioactive compounds, discovering new phytochemicals and discriminating medicinal plant species by their chemical “fingerprints”. Metabolomics is a useful tool for assessing chemotypic variations which are influenced by the environment (climate, soil composition, altitude and biotic stress) (Wenlong et al., 2023). In addition, metabolomic data can also be used for quality control, authentication and standardization of herbal medicines, providing characteristic metabolite markers. Metabolite annotation and pathway prediction have been further improved by recent advances in bioinformatics and ML, leading to a deeper understanding of secondary metabolite biosynthesis. Thus, metabolomics plays a fundamental role in natural product research, drug discovery, and evidence-based herbal therapeutics (Figure 2).

**FIGURE 2:**
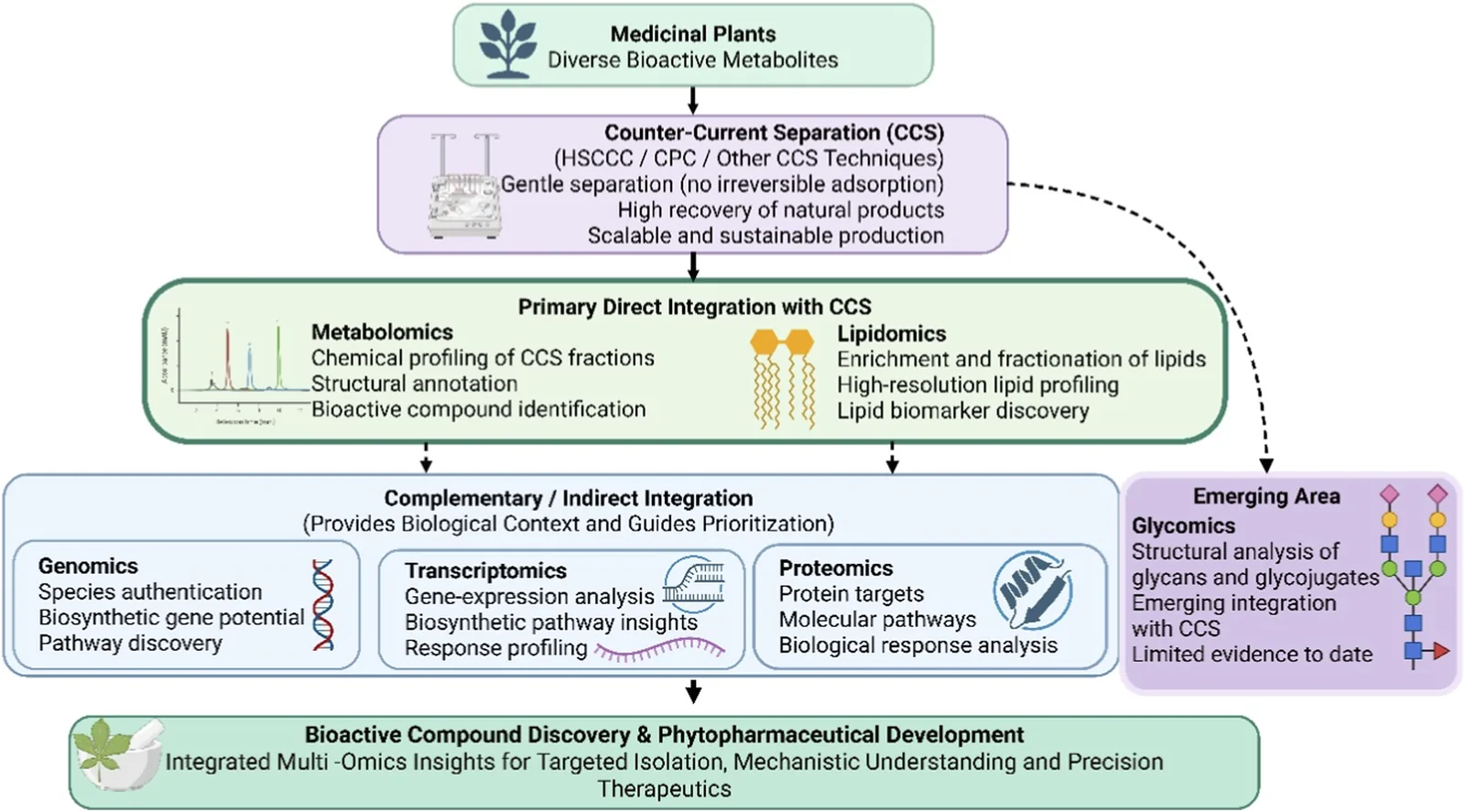
Hierarchical framework illustrating differential modes of interaction between counter-current separation (CCS) and omics technologies in medicinal plant research.

#### 4.9.2 CCS-omics integration is needed

Although natural product research has been revolutionized by CCS and omics technologies, their combination has only recently been developed as an integrated strategy to accelerate the discovery of bioactive metabolites. CCS uses only liquid-liquid partitioning and not a solid stationary phase; therefore, it allows for the fractionation of complex extracts with good recovery without irreversible adsorption (Ito and Ma, 1996). This is particularly useful for unstable, highly polar, or low-abundance metabolites that This approach is especially helpful when dealing with unstable, highly polar or low abundance metabolites that are typically lost during conventional chromatographic purification. In conventional chromatographic purification. However, CCS alone cannot yield precise structural annotation or systems-level biological interpretation. Metabolomics, proteomics, transcriptomics, and other omics platforms, on the other hand, generate comprehensive molecular datasets but often suffer from sample complexity and ion suppression problems when crude extracts are directly analyzed. CCS combined with omics technology solves these limitations by decreasing the sample complexity prior to high-resolution analytical profiling for metabolite annotation, biomarker discovery, and biological interpretation (Sun et al., 2020).

#### 4.9.3 Differential levels of integration between CCS and omics platforms

Importantly, the relationship between counter-current separation (CCS) and individual omics technologies is not equivalent and should not be interpreted as implying a direct analytical coupling for all omics domains. At present, the most direct and experimentally established integration is observed between CCS and metabolomics, including LC-MS-, MS/MS-, and NMR-based chemical profiling of CCS-generated fractions. Lipidomics may also be directly compatible with CCS when the objective is the enrichment or fractionation of lipid constituents prior to high-resolution analysis. In contrast, genomics and transcriptomics generally do not involve CCS as a direct analytical step; rather, these approaches provide complementary biological information, such as species authentication, biosynthetic gene identification, gene-expression patterns, and pathway reconstruction, which can subsequently guide the selection, prioritization, or biological interpretation of metabolites isolated using CCS. Similarly, proteomics is more appropriately positioned as a downstream or parallel approach for investigating protein expression, molecular targets, or biological responses associated with purified compounds or bioactive CCS fractions. The integration of glycomics with CCS remains comparatively less developed and should therefore be regarded as an emerging application requiring further methodological validation (Yiu et al., 2025).

Accordingly, the term “CCS–multi-omics integration” in this review refers to different levels of interaction between separation and omics technologies, ranging from direct analytical integration to indirect biological guidance and downstream mechanistic interpretation. These relationships should therefore be considered complementary rather than methodologically equivalent.

#### 4.9.4 CCS coupled with metabolomics

For CCS, metabolomics is the most applied omics tool since it allows the qualitative and quantitative description of metabolites within the CCS fraction (Kaśkosz et al., 2023). HSCCC or CPC fractions are usually analyzed by UHPLC-QTOF-MS, Orbitrap-MS or NMR spectroscopy to obtain detailed metabolite fingerprints. CCS pre-fractionation improves the detection of minor components, ionization efficiency and matrix interference compared with directly analyzing crude extracts. This strategy enables chemometric and molecular networking studies to quickly dereplicate and prioritize fractions containing unknown bioactive metabolites. Thus, CCS-guided metabolomics is a powerful approach for detection of pharmaceutically active naturally occurring chemicals without their isolation. Recent studies showed practical benefits from CCS and metabolomics. CPC-assisted fractionation and LC-MS-based metabolomics in medicinal plant phytochemical research Reduced sample complexity and identified bioactive flavonoids and phenolic acids. HSCCC and untargeted metabolomics were used for dereplication of curcuminoids, terpene lactones and flavonoid glycosides from Ginkgo biloba and Curcuma longa. CCS fractionation with high-resolution metabolomics allowed researchers of marine natural products to identify secondary metabolites of distinctive structures better than crude extracts alone. The data demonstrate that CCS-metabolomics approaches dramatically improve analytical sensitivity, compound recovery and confidence in metabolite identification (Schrimpe-Rutledge et al., 2018).

#### 4.9.5 Integration with proteomics and transcriptomics

Metabolomics is the most developed application of CCS, but it can be used in proteomics and transcriptomics to explore the molecular processes of bioactive compounds Medicinally active proteins in phytochemically rich CCS fractions can be identified by quantitative proteomics. Transcriptome studies of the isolated fractions show altered gene expression profiles pointing to a similar association between chemical composition and biological response. Combining transcriptome, proteome and metabolomic data sets improves target identification and understanding of pharmacological action systems. Integrated multi-omics approaches will have an ever-increasing role in evidence-based natural product research (Trivedi et al., 2026). While CCS may have a future, integration with omics technology presents significant challenges. Analytical throughput is limited as empirical optimization is still necessary to select biphasic solvent systems. Complex bioinformatic systems are needed to analyze multi-omics datasets from CCS fractions that may include transcriptome, proteome and metabolomic data (Table 8). However, the widespread applications are limited by the lack of defined analytical methods, software platform compatibility, and experimental confirmation of computer predictions. Future studies should standardize the processing of multi-omics data, automate the selection of solvents, annotate metabolites with AI, and integrate network pharmacology to improve reproducibility and translational applications.

**TABLE 8:** Representative studies demonstrating the integration of CCS with omics technologies for may improve the efficiency of natural product discovery, metabolite profiling, biomarker identification, and bioactivity-guided fractionation.

Natural source	CCS technique	Omics platform	Major application/findings	Advantages of CCS-omics integration	References
*Ginkgo biloba* leaves	HSCCC	LC-MS-based metabolomics	Isolation and annotation of flavonoids and terpene lactones for quality assessment	Improved metabolite resolution, reduced matrix interference, enhanced biomarker identification	Wolfender et al. (2019a)
*Curcuma longa* rhizomes	HSCCC	UHPLC-QTOF-MS metabolomics	Fractionation and characterization of curcuminoids and minor diarylheptanoids	High recovery of thermolabile metabolites with accurate metabolite profiling	Wolfender et al. (2019b)
Marine sponge extracts	CPC	LC-HRMS metabolomics	Discovery of novel marine secondary metabolites through bioactivity-guided fractionation	Efficient dereplication and prioritization of bioactive fractions	Gaudry et al. (2023)
Traditional Chinese Medicine formulations	CPC	Untargeted metabolomics + chemometrics	Identification of quality markers (Q-markers) and chemical fingerprints	Enhanced quality control and authentication of herbal formulations	Wolfender et al. (2019c)
Medicinal plant extracts	HSCCC	Molecular networking (GNPS) + LC-MS/MS	Rapid dereplication and prioritization of bioactive metabolites	Reduced redundancy in isolation and accelerated compound identification	Gaudêncio et al. (2023)
Plant bioactive fractions	CPC	Proteomics	Identification of protein targets associated with purified phytochemicals	Linked chemical composition with biological response	Hasin et al. (2017)
Botanical extracts	HSCCC	Transcriptomics (RNA-Seq)	Evaluation of gene expression changes induced by enriched phytochemical fractions	Mechanistic understanding of pharmacological activity	Sindhu et al. (2021)
Medicinal plants	HSCCC/CPC	Multi-omics (Metabolomics + Proteomics + Transcriptomics)	Systems-level investigation of bioactive natural products and mechanism elucidation	Comprehensive characterization and target validation	Queiroz et al. (2024)

#### 4.9.6 Proteomics in phytochemical research

Proteomics analyses organism-expressed proteins at scale. It helps explain medicinal plant secondary metabolite biosynthesis molecular mechanisms. Proteins act as enzymes, transporters, receptors, and regulators, hence proteomic research can help identify the most significant biological processes in pharmacologically active chemical production. TMT-based proteomics, two-dimensional gel electrophoresis (2-DE), LC-MS/MS, and isobaric tags for relative and absolute quantification (iTRAQ) can identify and quantify thousands of proteins at high throughput. In medicinal plants, proteomics is used to explore how environmental challenges, elicitors, and developmental phases affect phytochemical synthesis (Chele et al., 2025). Stress-responsive proteins can induce flavonoids, alkaloids, and terpenoids biosynthesis in response to drought, salinity, and microbial elicitors. Comparative proteomics can also discover proteins up- or downregulated in high- and low-metabolite-producing variants. Proteomic data might also be combined with transcriptomic and metabolomic data to confirm that gene expression changes produce functional proteins. Combining proteomics data with other omics data helps rebuild complicated metabolic networks, enabling metabolic engineering tactics to increase pharmaceutical product production (Wang et al., 2022b).

### 4.10 Transcriptomics and biosynthetic pathway analysis

Transcriptomics examines all RNA transcripts in a cell, tissue, or organism under a physiological situation. Therapeutic plant studies are necessary for transcriptome investigations of secondary metabolite production genes. High-throughput sequencing technologies like RNA sequencing (RNA-Seq) have revolutionised the discovery of biosynthetic genes and regulatory networks for the manufacture of pharmacologically relevant chemicals (Guo et al., 2025). Compared gene expression levels can show how environmental influences, developmental phases, and biotic interactions affect metabolite levels. Transcriptomic methods have revealed alkaloids, flavonoids, terpenoids, and phenylpropanoids biosynthesis pathways in numerous therapeutic plants. Endophytic fungi and microbial symbionts can increase secondary metabolite production by activating defense-related signal transduction pathways including jasmonic acid and salicylic acid (Guo et al., 2023). Transcriptomic analysis discovers transcription factors that control biosynthetic pathways, which can be targeted for metabolic optimisation and genetic alteration. Transcriptomics data can be utilised alongside metabolomics to connect gene expression with metabolite abundance, showing gene-phytochemical functional correlations. This system-level knowledge improves pathway reconstruction, synthetic biology, and scalable pharmaceutical chemical synthesis (Wang H. et al., 2024).

### 4.11 Genomics and DNA barcoding of medicinal plants

Genomes provide the genetic foundation for researching medicinal plant biological and chemical diversity. NGS and third-generation sequencing have increased the quantity of high-quality reference genomes for many medicinal species. Genomic data reveals secondary metabolite production, illness resistance, environmental adaptation, and evolutionary links genes. DNA barcoding, which employs short, standardised DNA sequences to identify medicinal plants, is a major genomics application in pharmacognosy (Heinrich and Jalil, 2023). Common barcode regions like rbcL, matK, ITS, and psbA-trnH can distinguish species even without morphology or after processing. DNA barcoding prevents adulteration, substitution, and contamination by identifying herbal product species with a short, standardised DNA sequence, enhancing quality assurance and consumer safety (Wang M. et al., 2024). WGS can also find phytochemical-producing biosynthetic gene clusters. Comparative genomics can identify plant metabolic pathway evolution and diversification. Genomic databases allow marker-assisted breeding and genome-editing to boost medicinal plant yield and phytochemical content. Thus, genomics is essential for high-level omics-based medicinal plant investigations (Yu et al., 2021b).

### 4.12 Lipidomics and glycomics in plant metabolism

Metabolomics subfields lipidomics and glycomics analyse lipids and glycoconjugates in detail. These approaches reveal the intricacy of metabolic pathways and the variety of bioactive chemicals in medicinal plants. Advanced analytical methods including LC-MS, GC-MS, and HRMS are utilised in lipidomics to characterise phospholipids, glycolipids, sterols, fatty acids, and signalling lipids (Buedenbender et al., 2020). Plant lipids have antibacterial, anti-inflammatory, antioxidant, and anticancer effects. Lipidomic studies help explain membrane dynamics and stress-adaptation mechanisms that regulate secondary metabolite synthesis. Glycomics examines the composition, structure, and biological activity of carbohydrates and glycoconjugated compounds such saponins, flavonoid glycosides, and polysaccharides. Glycosylation strongly affects phytochemical biological activity, solubility, stability, and bioavailability. Modern mass spectrometric and chromatographic methods make glycan molecules easier to analyse due to their structure and variety (Skalicka-Woźniak and Garrard, 2014). Lipidomic, glycomic, transcriptomic, and proteomic data will illuminate plant metabolism and find new medicinal drugs and metabolic indicators.

### 4.13 Multi-omics integration in ethnopharmacology

In ethnopharmacology, multi-omics integration is a new methodology that integrates the knowledge derived from genomics, transcriptomics, proteomics, metabolomics, lipidomics, and glycomics to create a comprehensive picture of medicinal plant systems. Traditional medicinal knowledge is a useful tool for identifying plants with therapeutic value, but the molecular mechanisms underlying their efficacy are often unknown (Table 9) (Chele et al., 2025). Multi-omics techniques relate genetic and metabolic pathways to pharmacologically active metabolites. Integrated analyses can authenticate medicinal species using genomics, identify biosynthetic genes using transcriptomics, verify protein expression using proteomics, and characterise bioactive chemicals using metabolomics. These rich data can be utilised to develop biological networks that show how environmental conditions, microbial interaction, and genetics affect phytochemical synthesis (Godisela and Balaraju, 2026). Multi-omics techniques have confirmed ethnopharmacological uses of numerous medicinal plants and linked therapeutic activity to molecular processes. AI and advanced bioinformatics tools expedite biomarker discovery and pathway prediction by integrating and analysing high-throughput omics data (Figure 3). A systems biology perspective supports drug discovery, quality control, conservation, and metabolic engineering. Thus, multi-omics integration is vital for turning conventional medicinal knowledge into scientifically validated and therapeutically applicable therapeutic resources (Ivanisevic and Sewduth, 2023).

**TABLE 9:** Differential modes of interaction between CCS and omics technologies in medicinal plant research.

Omics technology	Primary analytical role	Relationship with CCS	Level of integration	References
Metabolomics	Comprehensive chemical profiling	CCS fractionation directly followed by metabolomic analysis	Direct and well established	Rakusanova and Cajka (2024)
Lipidomics	Characterization of lipid species and lipid biomarkers	CCS may enrich or simplify lipid-containing fractions before analysis	Potentially direct; emerging/application-dependent	Al-Amrani et al. (2021)
Glycomics	Structural analysis of glycans and glycoconjugates	CCS may assist enrichment or fractionation of glycosylated compounds	Emerging and limited evidence	Wang H. et al. (2024)
Proteomics	Protein expression and target/pathway analysis	Usually applied downstream to investigate biological responses or targets associated with CCS-isolated compounds/fractions	Indirect/complementary	Waterworth (2025)
Transcriptomics	Gene-expression and biosynthetic pathway analysis	Transcriptomic information can guide metabolite prioritization or interpret biological effects of isolated compounds	Indirect/biologically guided	Rakusanova and Cajka (2024)
Genomics	Species authentication and biosynthetic potential	Genomic information supports plant authentication and biosynthetic pathway discovery rather than direct CCS analysis	Indirect/contextual	Barboza et al. (2021)
Metabolomics	Comprehensive chemical profiling	CCS fractionation directly followed by metabolomic analysis	Direct and well established	Wan et al. (2025)

**FIGURE 3:**
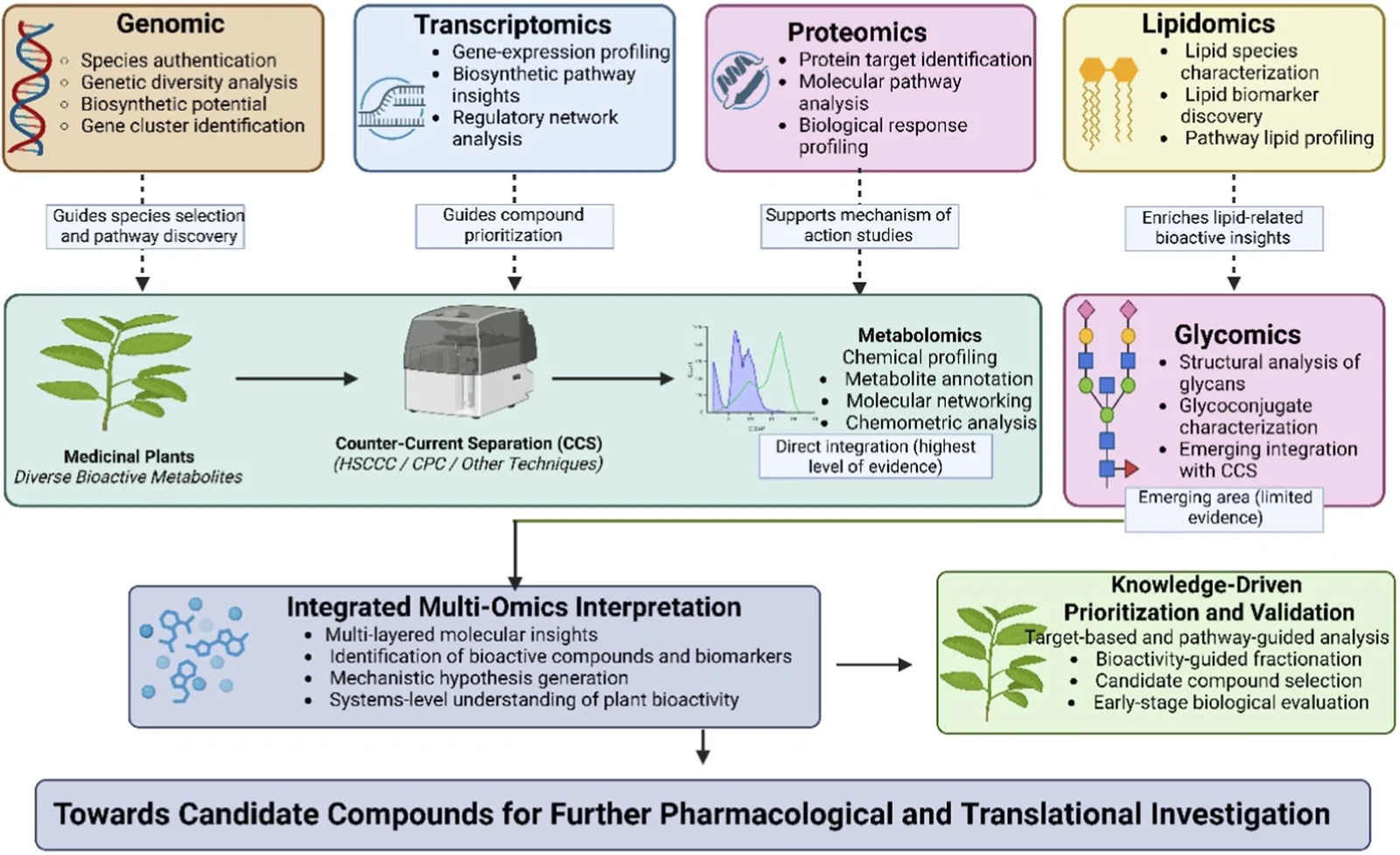
Operational integration of complementary omics information with CCS-based natural product research.

Multi-omics approaches have enhanced our knowledge about the pathways for biosynthesis of natural products, the diversity of metabolites, and molecular mechanisms. The integration of genomic, transcriptomic, proteomic, and metabolomic data sets is computationally demanding due to the heterogeneity of data formats, analytical platforms, and biological diversity. Complex bioinformatic analyses, biological interpretation, expensive experiments, and lack of standardization of analytical procedures hinder broad acceptance. Computer forecast accuracy and lab reproducibility are still challenges. Future growth will be driven by standardized multi-omics pipelines, robust bioinformatic platforms, and AI-enabled data integration for biologically meaningful insights (Balotf et al., 2025).

## 5 Integration of CCS with omics platforms

### 5.1 CCS-metabolomics integration for compound discovery

Over the last few years, the combination of CCS and metabolomics has emerged as a powerful tool for may improve the efficiency of natural product discovery and phytochemical characterization. CCS techniques such as HSCCC and CPC enable preparative fractionation of complex plant extracts without a solid stationary phase. The fractions can then be further analyzed using metabolomics techniques such as UHPLC-MS/MS, GC-MS or NMR (Zhai et al., 2022). This is a good way to reduce matrix complexity and improve detection of metabolites present at low concentrations, which are usually not detected in crude extracts. CCS produces chemically enriched fractions, which leads to improved metabolites annotation, biomarker discovery and structural characterization. The integration also enables the discovery of new bioactive molecules, quality control of herbal formulations, and the acceleration of the drug discovery process (Figure 4) (Messaili et al., 2020). Therefore, CCS-metabolomics platforms can serve as a powerful tool for correlating phytochemical diversity with biological activity/therapeutic potential.

**FIGURE 4:**
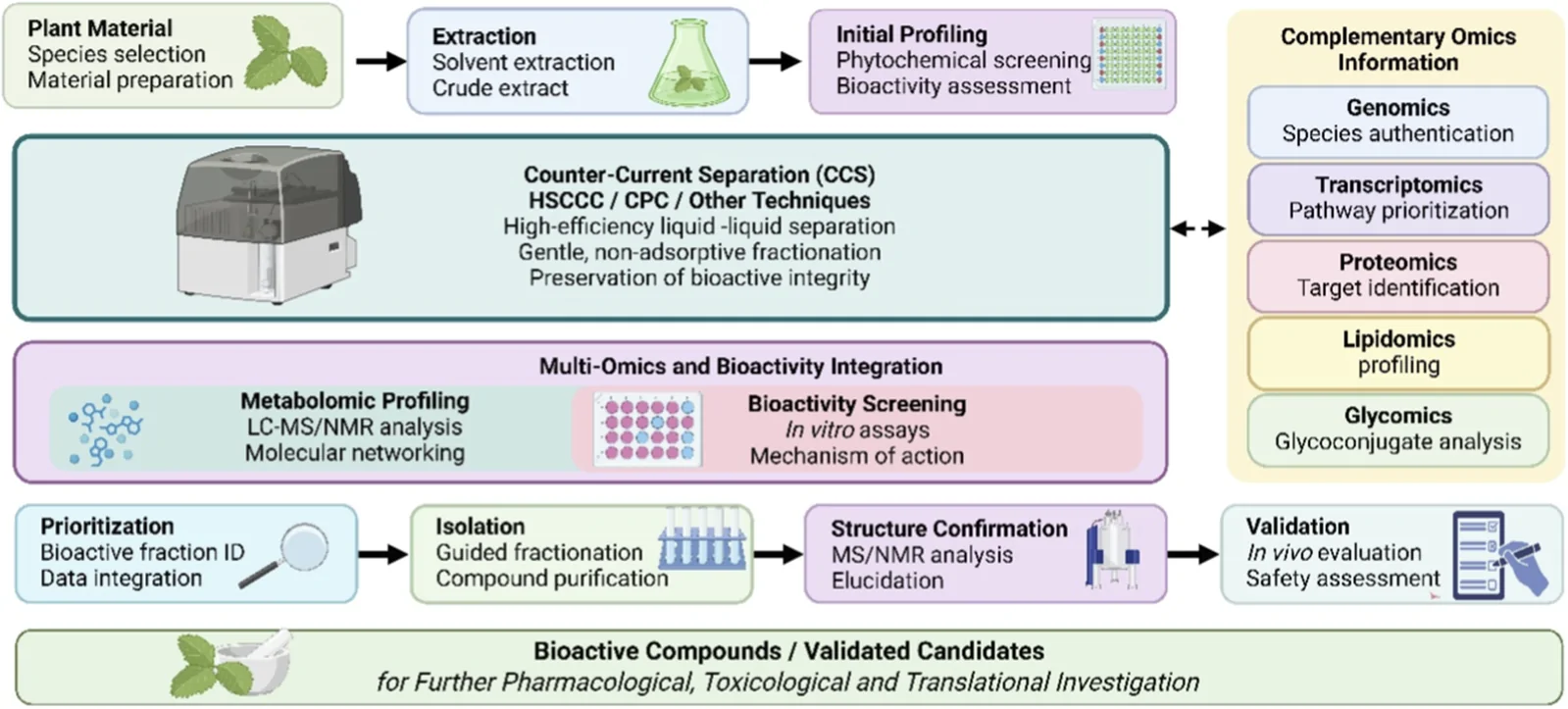
Integrated CCS-based compound discovery and bioactivity-guided prioritization workflow.

### 5.2 Dereplication strategies in natural product research

Coupling of CCS with cutting-edge analytical techniques such as LC-MS/MS and GC-MS enhances the efficiency of natural product research. CCS is an integrated workflow used as a preparative separation technique to reduce sample complexity before spectrometric analysis. LC-MS/MS then analyses the fractions produced for non-volatile components, and GC-MS analyses those for volatile and semi-volatile constituents. This hyphenated technique allows the identification, characterization, and quantification of phytochemicals found in complex botanical extracts in a short time. Accurate molecular weight determination, fragmentation patterns and structural information provided by HRMS can speed up the dereplication and compound annotation process (Gaudêncio et al., 2023). In addition, the integration reduces the effects of coeluting compounds and enhances analytical sensitivity. The advantage of combining CCS with mass spectrometry is that it enables efficient identification of novel bioactive molecules and shortens the process of natural product isolation.

### 5.3 CCS-coupled NMR and structural elucidation

The combination of CCS and NMR is a very useful tool for the structural elucidation of natural products. The CCS techniques can be used to obtain highly purified fractions, usually above 95% purity, suitable for detailed spectroscopic investigations. Because CCS has no stationary phase, isolated compounds are not contaminated by silica particles, column bleed or degradation products of the stationary phase (Seger and Sturm, 2022). This leads to NMR spectra of unsurpassed purity and clarity, with minimal background interference. Isolation of metabolites allows for detailed structural analysis using advanced NMR techniques such as ^1H-NMR, ^13C NMR, COSY, HSQC, HMBC and NOESY. The molecular connectivity, functional groups, stereochemistry and three-dimensional configurations can be determined with high accuracy. The CCS-NMR workflow can be very useful for the identification of new natural products, the structure elucidation of molecules and the validation of bioactive compounds from medicinal plants, marine orogenic and microbial sources (Chontzopoulou et al., 2025).

### 5.4 Chemometric and bioinformatic approaches

The large and complex datasets generated by integrated CCS and omics platforms are integral to their interpretation, and chemometric and bioinformatic tools are essential in this regard. There are several techniques that have been widely used to identify meaningful patterns in multidimensional data, such as Principal Component Analysis (PCA), Hierarchical Cluster Analysis (HCA), Partial Least Squares Discriminant Analysis (PLS-DA), and Orthogonal Partial Least Squares Discriminant Analysis (OPLS-DA) (Ma et al., 2021). These statistical methods allow samples to be classified, biomarkers to be identified, and differences in the phytochemical composition of species, cultivars, or processing conditions to be distinguished. Bioinformatics platforms also facilitate metabolite annotation, pathway analysis, and the construction of molecular networks. A metabolomic profile of a medicinal plant extract can be produced using CCS fractionation and compared with biological activity to correlate specific metabolites with biological activity (He et al., 2025). The combination of chemometrics and bioinformatics in data interpretation, decision-making in fraction selection, and understanding complex phytochemical and biological relationships will be enhanced.

### 5.5 AI and ML in omics-integrated CCS

The advent of AI and ML is transforming the integration of CCS with omics technologies by enabling predictive, data-driven analytical workflows. Optimizing conventional CCS solvent systems typically requires numerous experiments and is time-consuming and labor-intensive. ML algorithms can be used to extract information from large datasets of molecular descriptors, partition coefficients, physicochemical properties, and chromatographic parameters to predict optimal solvent systems accurately (Kalpana and Prasad, 2024). Neural networks and other advanced deep learning models can discover complex nonlinear relationships between compound partitioning and separation efficiency. AI tools also assist in metabolite annotation, spectral interpretation, biomarker discovery, and molecular network analysis. In addition, models can be used for guiding the prioritization of fractions and natural product isolation strategies. The combination of AI, omics and CCS data allows researchers to improve separation performance, speed up compound identification, better reproduce pharmaceutical and phytochemical studies and improve research efficiency (Vaškevičius et al., 2021).

### 5.6 Distinguishing compound discovery from drug development

It is important to distinguish bioactive compound discovery from drug discovery and subsequent therapeutic development. CCS-assisted fractionation, metabolomic profiling, molecular networking, and other omics methods can help find, rank, isolate, and provide a first look at candidate natural products. Bioactivity-guided experiments may further establish associations between specific compounds or fractions and measurable biological effects. However, these stages represent early discovery and validation activities and do not, by themselves, establish a compound as a drug candidate or therapeutic product. Progression toward lead optimization or botanical drug development requires additional evidence, including reproducible pharmacological activity, mechanism-of-action studies, dose–response characterization, safety and toxicological assessment, pharmacokinetic evaluation, formulation and manufacturing development, regulatory assessment, and, where appropriate, clinical investigation. Accordingly, the role of CCS–omics integration should be interpreted primarily as enabling compound discovery and early-stage biological characterization rather than as directly producing new drugs or future botanical therapeutic development (Allen et al., 2026).

### 5.7 Challenges, limitations, and future perspectives of CCS-Omics integration

The combination of CCS and multi-omics technologies is a great contributor to the research of natural products (Avitabile et al., 2024). But its wider use is still hampered by some major technical and practical limitations. One of the major challenges is the integration of multi-omics data from genomics, transcriptomics, proteomics, metabolomics and lipidomics (Huang et al., 2023). The datasets differ significantly regarding data structure, analytical resolution, normalization and biological variability and the in-depth interpretation is computationally demanding (Mayer, 2011). Several existing bioinformatic approaches require lengthy pre-processing, imputation of missing values, correction of batch effects and complex statistical modeling before any meaningful biological conclusion can be drawn (Ahsan et al., 2022). Another major problem is the lack of modern analytical instruments. The complete CCS-omics platforms are developed based on HSCCC or CPC systems combined with high-resolution LC-MS/MS, GC-MS, NMR spectroscopy and high-performance computing capacity. Sanches, P. H. G. 2023. These instruments are also expensive to buy, maintain, calibrate and use and so these are mainly seen in well-funded academic institutions and specialized research laboratories. The demand for multidisciplinary knowledge in analytical chemistry, computational biology, bioinformatics and ML is another hurdle for routine applications (Table 10).

**TABLE 10:** Critical comparison of CCS–omics integration with conventional natural-product discovery workflows.

Parameter	Conventional chromatography	CCS	CCS + omics	Critical interpretation	References
Sample recovery	Moderate	High	High	CCS minimizes irreversible adsorption	Brent and Pauli (2005)
Structural annotation	Limited after fractionation	Limited	High	Omics improves metabolite annotation	Wolfender et al. (2019c)
Sample complexity	High	Reduced	Strongly reduced	CCS pre-fractionation improves MS analysis	Berthod and Sydow (2023)
Biomarker discovery	Limited	Moderate	High	Omics provides molecular-level information	Goodacre et al. (2023)
Solvent optimization	Conventional optimization	Often laborious	AI-assisted potential	AI remains insufficiently validated	Wei et al. (2026)
Data complexity	Low–moderate	Moderate	Very high	Multi-omics requires advanced bioinformatics	Hayes et al. (2024)
Reproducibility	Method dependent	Method dependent	Potentially high	Standardization remains a major limitation	Wolfender et al. (2019c)
Scale-up	Established	Feasible	Still developing	Industrial validation is required	Armstrong and Berthod (2023)
Regulatory readiness	Established	Developing	Emerging	Standardized validation is needed	Goodacre et al. (2023)
Cost	Moderate–high	High	Very high	Instrumentation and data analysis increase costs	Dorling et al. (2025)

Standardization is another big problem. There is a high diversity between laboratories in terms of solvent system choice, CCS operating parameters, omics sample preparation, data pre-processing, metabolite annotation and statistical analysis pipelines (Liu X. et al., 2015). This methodological heterogeneity makes it difficult to compare studies and hampers reproducibility. Moreover, the generalizability of the identified biomarkers and algorithms is restricted as many of the published CCS-omics studies are limited by small sample sizes and lack external validation with other datasets. However, the integration of CCS-omics has many unique benefits over traditional methods that might make natural product discovery methods more efficient. Labile metabolites can be high-yield recovered by liquid-liquid partitioning with CCS without the problem of irreversible adsorption on solid stationary phases. CCS and multi-omics strategies combined allow the full characterization of complex phytochemical systems, acceleration of biomarker discovery and dereplication and systems-level biological interpretation. Continued developments in AI, automated data integration pipelines, standardized reporting guidelines, cloud-based bioinformatics platforms, and multicenter validation studies will overcome existing challenges and increase the robustness, reproducibility, and clinical translation of CCS-assisted natural product research (Ciotta et al., 2025).

### 5.8 Critical appraisal of CCS–omics integration

#### 5.8.1 Scientific strengths of CCS–omics integration

The synergy of counter-current separation (CCS) and omics technologies provides major scientific benefits in the study of complex natural product matrices. High-speed counter-current chromatography (HSCCC) and centrifugal partition chromatography (CPC) are CCS methods that allow efficient liquid–liquid fractionation without a solid stationary phase, thus minimizing irreversible adsorption and improving the recovery of chemically diverse and potentially labile metabolites. Fractionation before LC-MS/MS, GC-MS, or NMR analysis can reduce matrix complexity and ion-suppression effects, improve metabolite detection, and facilitate the characterization of minor constituents. In addition, the high recovery and preservation of bioactive molecules provided by CCS can increase the availability of material for subsequent structural elucidation and biological evaluation (Frolova et al., 2025).

A further strength of CCS–omics integration is its ability to connect chemical composition with biological activity. CCS-generated fractions can be subjected to metabolomic profiling and bioactivity-guided screening, while chemometric analysis, molecular networking, and bioinformatic approaches can be used to associate specific metabolites or molecular clusters with observed pharmacological effects. This integrated strategy improves dereplication, facilitates prioritization of bioactive fractions, and can reduce unnecessary repeated isolation of previously characterized compounds. The studies summarized in the manuscript have shown that CCS combined with metabolomics can improve the resolution of metabolites, reduce the matrix interference, improve the identification of biomarkers, and facilitate the discovery of bioactive natural products. However, these advantages still rely on the proper conditions of fractionation, capabilities of the analytical platform, annotation of metabolites, and final validation of results (Gaudêncio et al., 2023).

#### 5.8.2 Methodological weaknesses

Despite the advantages of CCS–omics integration, several methodological limitations restrict its broader application. A major limitation is that biphasic solvent-system optimization is largely empirical and can be time-consuming. Solvent composition, partition coefficients, phase ratios and other operating parameters must be carefully optimized for individual sample matrices. Moreover, the variable retention of the stationary phase during the operation of CCS might influence the separation efficiency and reproducibility, especially when the composition of the solvent system or the operating conditions are not well controlled. Poor resolution can also be observed with compounds having similar partition coefficients. In this case the distribution of the compound between the two liquid phases is similar, and it is difficult to separate structurally related metabolites. These limitations indicate that CCS performance remains highly dependent on compound physicochemical properties and optimized experimental conditions (Wang et al., 2022c).

Another important limitation is the high instrumentation and operational cost associated with integrated CCS–omics workflows. Comprehensive workflows may require HSCCC or CPC systems combined with high-resolution LC-MS/MS, GC-MS, NMR spectroscopy, and advanced computational infrastructure, resulting in substantial costs for equipment acquisition, maintenance, calibration, and operation. Furthermore, integration of metabolomics, transcriptomics, proteomics, and other omics datasets introduces considerable computational complexity because of differences in data formats, analytical resolution, normalization procedures, batch effects, missing-value handling, and metabolite annotation strategies. These requirements also demand multidisciplinary expertise in analytical chemistry, bioinformatics, computational biology, and machine learning. Consequently, although CCS–omics integration provides a powerful platform for natural-product research, its reproducibility, accessibility, and scalability remain constrained by methodological optimization, instrumentation requirements, data-processing complexity, and the need for standardized workflows (Jiang et al., 2025).

#### 5.8.3 Evidence limitations

The application of CCS–omics to natural-product research is growing, but the evidence base remains constrained by the predominance of small studies and a lack of external validation. Many reported workflows are developed and evaluated with individual plant extracts, limited sample sets, or specific experimental conditions, limiting the generalizability of findings to other botanical sources and laboratories. In addition, computational models and biomarker predictions generated from CCS–omics datasets are frequently not validated using independent datasets or experimentally confirmed biological systems. Consequently, reported associations between metabolites, molecular signatures, and biological activities should be interpreted cautiously until they are reproduced using larger and independently validated datasets (Table 11) (Hu and Jia, 2021).

**TABLE 11:** Evidence-based assessment of AI maturity in CCS.

AI application	Current status	Evidence level	Main limitation
Spectral preprocessing	Established	High	Dataset dependence
Peak detection	Established	High	Platform variability
K-value prediction	Developing/established	Moderate	Dataset size
Solvent-system prediction	Emerging	Moderate	External validation
Deep-learning CCS optimization	Emerging	Low–moderate	Small datasets
Digital twins	Emerging	Low	Limited real-world validation
Autonomous CCS	Future	Low	Lack of validated workflows

A further evidence gap arises from limited cross-laboratory reproducibility, heterogeneous analytical platforms, and insufficiently standardized reporting practices. Differences in CCS solvent-system selection, operating parameters, sample preparation, omics platforms, data preprocessing, metabolite annotation, and statistical analysis can produce variations between studies and make direct comparison difficult. The paper also highlights that the lack of standardized analytical methods, software compatibility, and experimental validation of computational predictions are major obstacles to a broader application. Consequently, future CCS–omics studies should focus on standardized experimental and reporting methods, well-defined quality-control metrics, interoperable data formats, independent validation cohorts, and cross-laboratory reproducibility studies. Such steps are critical to the translation of promising proof-of-concept results into reliable and reproducible analytical workflows suitable for pharmaceutical and natural-product investigations (Rampler et al., 2021).

#### 5.8.4 Translational limitations

Although CCS–omics integration provides a powerful platform for natural-product discovery, the identification of metabolites or bioactive chemical signatures does not by itself establish therapeutic efficacy. Metabolomic profiling and dereplication can prioritize candidate compounds and bioactive fractions, but these findings require subsequent pharmacological investigation to establish mechanism of action, dose–response relationships, efficacy, and safety. Similarly, metabolite annotation should not be considered equivalent to definitive structural confirmation. Metabolite identities proposed by MS/MS databases or computational annotation should be confirmed with authentic standards and complementary techniques such as NMR or other orthogonal structural techniques. Thus, the transition from metabolite discovery to a validated therapeutic lead requires sequential chemical, biological, and pharmacological confirmation (Maan et al., 2025).

The need for experimental validation of AI/ML predictions and standardized quality and manufacturing processes also constrains the translational potential of CCS–omics workflows. Artificial intelligence can help to choose the solvent system, annotate metabolites, prioritize biomarkers, and optimize chromatography, but predictive models must be tested on independent datasets and experimentally validated before they can be used to reliably inform decision-making. Furthermore, the biological activity found in fractions from CCS must be confirmed by appropriate pharmacological and toxicological studies before any therapeutic claims. Regulatory requirements for botanical or phytopharmaceutical products include reproducible botanical authentication, chemical characterization, quantification of marker compounds, batch-to-batch consistency, validated analytical procedures and appropriate GMP controls. The manuscript describes the reporting principles and regulatory framework (FDA and EMA guidance) for ConPhyMP to enhance transparency, reproducibility, quality control and regulatory evaluation (Su et al., 2026).

## 6 Applications in bioactive compound discovery

### 6.1 Isolation of alkaloids and nitrogenous metabolites

However, owing to the strong interaction between basic nitrogen and acidic silanol groups, traditional silica gel chromatography can be cumbersome for alkaloid purification. In contrast, pH-zone refining CCS can effectively overcome this issue. The rhizomes of Coptis chinensis were efficient extracted with a chloroform-methanol-water system, from which quaternary ammonium alkaloids (berberine, palmatine, coptisine, jateorhizine and columbamine) were isolated (Nakonieczna et al., 2022). The stationary phase was modified with a retentate (hydrochloric acid), and the mobile phase with an eluate (triethylamine). This displacement mode resulted in very high purities (typically >98%) and very high sample throughput directly from crude extracts. In addition, dangerous lipo-alkaloids and monoester-diterpenoid alkaloids from “Kusnezof monkshood root” have been successfully fractionated into ultra-pure blocks, using the same gradient methods (Zhao et al., 2020).

### 6.2 Purification of flavonoids and polyphenols

The polarity profile of flavonoids is very broad, and the glycosylation state is extremely important. Recently, DES extraction, when combined orthogonally with HSCCC, has proven highly synergistic for this class of compounds (Yu et al., 2021a). Choline chloride and 1,4-butanediol DES matrices were used to efficiently extract and purify specific flavonoids (isovitexin, padmatin and quercetin) from the complex thorns of Gleditsia sinensis, with tailored quaternary solvent systems (e.g., dichloromethane-methanol-n-butanol-water) that afforded resolutions unattainable by traditional prep-HPLC. CCS also routinely offers preparative isolation of unique flavonoid profiles from processed plants such as Daphne genkwa with high purity (Mi et al., 2023).

### 6.3 Discovery of terpenoids and essential oil constituents

The most difficult compounds for CC methods are terpenoids and lipophilic essential oils, which are almost exclusively partitioned into the non-polar organic phase. Advanced CCS techniques employ physical compression of the phase volume ratios or a completely non-aqueous binary system to shift *K* values back into the operational sweet spot. High-resolution HSCCC can be used in different modes, including overlapping elution-extrusion, to effectively separate highly complex isomeric monoterpenes with slight stereochemical differences in such products as Paeoniae Alba Radix (Marques et al., 2024; Wang B. et al., 2024).

### 6.4 Isolation of saponins and glycosides

Saponins are highly surfactant-like amphiphilic triterpene (or steroid) glycosides. Saponins are commonly cited as giving unbreakable emulsions, particularly during normal liquid-liquid extraction. HSCCC counteracts these hydrodynamic instabilities by continuous high-g centrifugal mixing. By using a highly surface-active tert-butyl methyl ether-butanol-acetonitrile-water (TBME-BuOH-ACN-H2O 1:2:1:5) system, researchers were able to separate highly surface-active acetal-type and dioxolane-type saponins from Beta vulgaris. Interestingly, acetal-type saponins were eluted earlier than the dioxolane-type ones, laying the groundwork for a robust preparative procedure for further fragmentomic study by LC-MS/MS. The continuous liquid-liquid partitioning method has also been used to successfully process other complex steroid glycosides, such as shatavarin IX and asparagoside A (Skalicka-Woźniak and Garrard, 2014; Spórna-Kucab and Wybraniec, 2020; Jasim, 2021).

### 6.5 Marine and microbial natural products

Marine organisms and extremophile endophytic fungi offer a wide range of chemical diversity, providing a rich source of novel pharmacophores. CPC and HSCCC have been highly useful in isolating highly complex marine secondary metabolites. Superior purities of the multi-ring polyketide antibiotic JBIR-99, which is highly effective against Gram-positive as well as Gram-negative bacterial pathogens (Wu et al., 2019), were achieved by the direct purification from the solid-state fermentation of the marine fungus Meyerozyma guilliermondii in an optimized petroleum ether-ethyl acetate-95% ethanol-water (5:3:5:3) solvent system. Likewise, the neuroprotective phlorotannin eckmaxol was successfully isolated from the crude organic extract of the marine brown alga Ecklonia maxima by the n-hexane/ethyl acetate/methanol/water matrix (Table 12).

**TABLE 12:** Representative phytochemical classes isolated using CCS techniques, including major bioactive compounds, separation methods, source plants, and applications in may improve the efficiency of natural product discovery.

Phytochemical class	Representative compounds	CCS technique	Source plant	References
Alkaloids	Berberine, Coptisine, Palmatine	pH-Zone HSCCC	Coptis chinensis	Sun et al. (2014)
Flavonoids	Quercetin, Isovitexin	HSCCC	Gleditsia sinensis	Andrade et al. (2021)
Polyphenols	Chlorogenic Acid	CPC/HSCCC	Various medicinal plants	Wang et al. (2025)
Terpenoids	Monoterpenes	HSCCC	Paeoniae Alba Radix	Ding et al. (2025)
Saponins	Acetal-type Saponins	HSCCC	Beta vulgaris	Yang et al. (2021)
Marine Metabolites	Eckmaxol, JBIR-99	CPC/HSCCC	Marine algae and fungi	Pan et al. (2024)

### 6.6 Rare and trace bioactive metabolites

Iterative solid-phase fractionation steps often discard all trace metabolites. In this way, phytochemists can focus very small metabolites into very strong bands using pH-zone refining and continuous gradient elution modes. CCS enabled the successful concentration and isolation of trace amounts of caffeic acid and high-purity chlorogenic acid directly from complex crude botanical matrices without being destructive (Lu et al., 2013).

## 7 AI in CCS

### 7.1 Established applications of AI in CCS

In many established research areas, chromatography, spectral deconvolution, data pre-processing, peak detection, chromatographic fingerprint analysis, chemometric modeling, compound classification and AI have all been successfully applied. Some of the main applications of AI in CCS are K-value statistical modeling, biphasic solvent system optimization based on previous experimental datasets and chromatographic performance prediction based on physicochemical descriptors. The use of ML approaches in metabolomics research has also been applied to the dereplication and prioritization of bioactive fractions generated by CCS. Such applications are leading the way for AI in chromatographic analysis and experimental studies (Figure 5) support such applications which are leading the way for AI in chromatographic analysis. by experimental studies (Figure 5).

**FIGURE 5:**
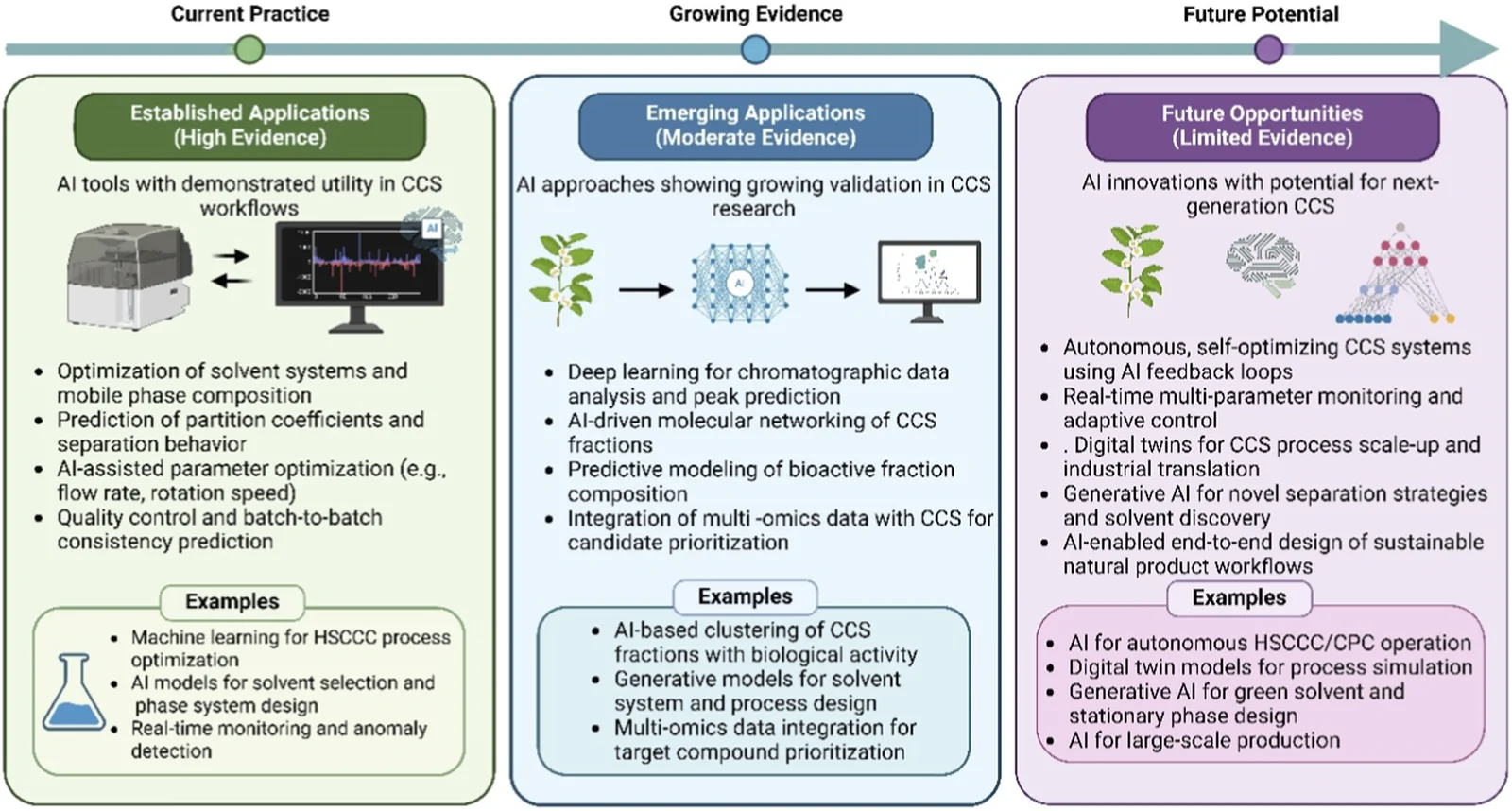
Evidence maturity of artificial intelligence applications in counter-current separation.

### 7.2 Emerging applications of AI in CCS

Many new AI-based applications have been proposed recently, although they are still under development. Deep learning approaches study adaptive CCS separation optimization, virtual chromatographic screening, optimal operating parameter prediction, and automated solvent system suggestion. Real-time chromatographic optimization has been investigated using digital twins, reinforcement learning, and autonomous lab platforms. Most of these studies were done on small datasets or in artificial environments and were not validated on natural product matrices. At present, these technologies represent promising new tools rather than proven analytical tools (Elagamy et al., 2026).

### 7.3 Future perspectives and challenges

Topol EJ said: “We will create AI-enabled CCS platforms with autonomous solvent selection, operational parameter optimization, real-time chromatographic performance monitoring, and linking of CCS outputs with metabolomics, molecular networking and network pharmacology analyzes.” Intelligent analytical platforms can save experimental time, improve reproducibility, and increase analytical throughput. But there are still major obstacles. Prediction models need external validation; algorithms are difficult to understand; regulatory approval is needed; and reproducibility in different labs and CCS datasets is not standardized. These challenges need to be addressed before AI-driven CCS can be used by pharmaceutical and natural product labs. However, many challenges have impeded the application of CCS-omics integration in pharmaceutical and natural product research, despite its success. Integration of genome, transcriptome, proteomic, metabolomic and lipidomic datasets is challenging. These datasets are difficult to interpret because of differences in analytical resolution, dynamic range, normalization approaches, data format, and biological variability. Due to substantial preprocessing, feature alignment, batch-effect correction, missing value imputation, and multidimensional statistical modelling, current bioinformatic pipelines cannot generate meaningful biological relationships (Li et al., 2026). Thus, multi-omics integration requires common data formats, interoperable databases, and strong computational frameworks. Another disadvantage is the verification of the accuracy of the AI forecast (Table 13).

**TABLE 13:** Current bottlenecks and future solutions for CCS-omics integration.

Current bottleneck	Scientific impact	Future strategy	References
Heterogeneous multi-omics datasets	Difficult data integration and biological interpretation	Standardized databases, interoperable workflows, AI-assisted integration	Wolfender et al. (2019c)
Validation of AI predictions	Limited clinical confidence	External validation, multicentre studies, explainable AI	van der Schaar et al. (2021)
Regulatory acceptance	Delayed translation of biomarkers	Standardized validation protocols, harmonized regulatory guidelines	European Medicines Agency (1994)
Industrial scalability	High operational cost and infrastructure requirements	Automation, cloud computing, high-throughput analytical platforms	Afrin et al. (2025)
Reproducibility	Inter-laboratory variability	Standard operating procedures and reference materials	Broadhurst et al. (2018)

Machine learning and deep learning approaches have also shown promise for predicting solvent systems, annotating metabolites, identifying biomarkers, and optimizing chromatography. However, many published models use retrospective research or small experimental datasets. To be integrated with confidence into analytical workflows, AI-assisted decision-making requires independent multicenter validation, prospective experimental verification, model transparency, and explainable AI techniques. Worse, you cannot even test prediction systems in a neutral way with performance indicators or benchmark datasets. Clinical and industrial applications of CCS-omics technology face regulatory approval limitations. For approval of integrated omics biomarkers for pharmaceutical research and precision medicine by regulatory agencies, clinical qualification, biological verification, and analytical validation are required. Omics-derived biomarkers require consistent reporting, quality assurance, and validation procedures to increase trust and to facilitate regulatory approval. Last but not least, the high cost of cutting-edge computational infrastructure, highly trained interdisciplinary staff, high-resolution mass spectrometry, nuclear magnetic resonance spectroscopy, and chromatographic instrumentation is preventing industrial adoption of CCS-omics integration for high-throughput, which may improve the efficiency of natural product discovery. Therefore, the future development should be oriented toward automated systems, cloud-based bioinformatics platforms, standardized analytical techniques, and low-cost high-throughput technologies that would improve scalability, reproducibility, and analytical robustness (Galal et al., 2022).

Despite AI’s promising future in CCS, there is currently only a modest body of evidence to support its use. Until now, most studies have focused on either retrospective modelling or proof-of-concept demonstrations rather than prospective validation using independent experimental data. A large number of categories of natural products still suffer from inadequate training data in terms of diversity and quality, which has a direct impact on forecast accuracy (Table 14). Consequently, existing AI-assisted CCS should be used with caution, and additional multicenter validation studies are required before these methods can be considered reliable analytical tools for routine use (Gangwal and Lavecchia, 2025).

**TABLE 14:** Current status of AI applications in CCS, distinguishing established applications, emerging developments, and future research opportunities.

AI application category	CCS application	Current level of evidence	Major limitations	Future opportunities	References
Established	Chemometric analysis of CCS fractions and metabolomic datasets	Widely applied in natural product metabolomics and chromatographic data interpretation	Requires high-quality curated datasets; model performance depends on data preprocessing	Automated feature extraction and improved biomarker discovery	Wolfender et al. (2019a)
Established	ML-assisted chromatographic fingerprint analysis	Experimentally validated for classification, authentication, and quality control of herbal medicines	Limited transferability across different analytical platforms	Integration with CCS-based quality assessment workflows	Gaudry et al. (2023)
Emerging	AI-assisted prediction of CCS solvent systems (partition coefficient, K value)	Early proof-of-concept studies; limited experimental validation	Small experimental datasets, limited generalizability, lack of benchmark databases	Data-driven solvent recommendation systems and automated method development	Raheem et al. (2026)
Emerging	AI-guided optimization of CCS operating parameters (flow rate, rotation speed, solvent composition)	Preliminary reports and simulation-based investigations	Few prospective experimental studies; no standardized validation	Autonomous optimization of CCS separations	Chi et al. (2024)
Future	Digital twin models for CCS process optimization	Conceptual; not yet implemented routinely	Lack of experimental datasets and validated predictive models	Intelligent process monitoring and real-time optimization	Topol (2019)
Future	Reinforcement learning and autonomous CCS platforms	Conceptual stage	No routine laboratory implementation; computational complexity	Fully automated CCS-omics-AI analytical platforms	Raghu and Schmidt (2020)

## 8 Bioactivity-guided fractionation and pharmacological evaluation

### 8.1 Integration of CCS with bioassay-guided screening

Unlike traditional bio-directed screening, CCS in combination with bioassay-guided screening has greatly enhanced the efficiency of natural product drug discovery. Standard bioassay-guided fractionation can involve repeated extraction, purification, and biological testing, leading to a lengthy, repetitive process and the potential for compound rediscovery. Complex extracts can be fractionated rapidly and with high recovery while retaining compound integrity using modern CCS methods such as HSCCC or CPC (Buedenbender et al., 2020). Recent developments have integrated Bioactive Molecular Networking (BMN), in which biological activity information is included directly with the LC-MS/MS-generated molecular networks. The biological activities (cytotoxic, antimicrobial, enzyme inhibition, etc.) are mathematically correlated with the spectral clusters, enabling the prioritization of specific families of molecules for isolation in this strategy (Figure 6). In this approach, the number of unnecessary purification steps is reduced, and active constituents are identified more quickly. Additionally, the use of CCS in conjunction with metabolomics, cheminformatics, and ML aids in the identification of novel bioactive compounds, hit prioritization, and boosts the likelihood of successful natural product studies for pharmacologically relevant applications (Mani et al., 2022).

**FIGURE 6:**
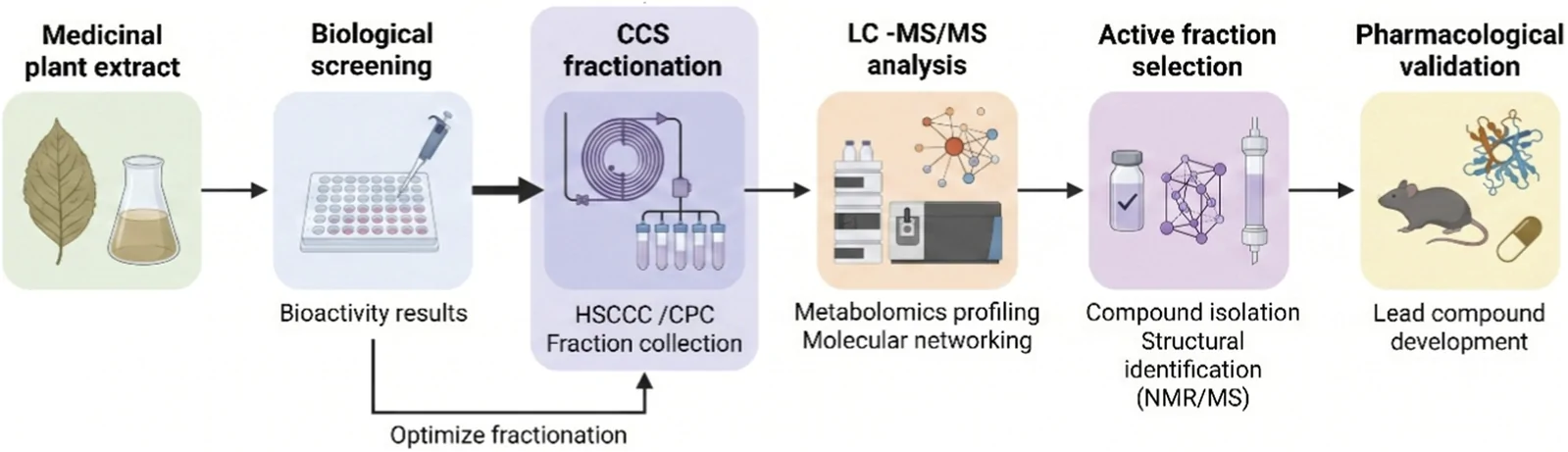
Workflow of CCS-based bioactivity-guided fractionation showing medicinal plant extract preparation, biological screening, HSCCC/CPC fractionation, LC-MS/MS metabolomics analysis, active fraction selection, compound isolation, structural elucidation, and pharmacological validation for lead compound development.

### 8.2 Anticancer natural products

Natural products remain a significant source of anticancer drugs, and isolation tools for the purification and evaluation of natural products based on the CCS principle are essential and invaluable. The purity of compounds produced by HSCCC and CPC is high, and they are often evaluated across many cancer cell lines to determine their antiproliferative mechanisms. Medicinal plants have been studied for their extracts, which contain various natural compounds that exhibit significant anticancer activity by modulating several cellular pathways, including alkaloids, flavonoids, terpenoids and polyphenols (Nakonieczna et al., 2022). In particular, coptisine extracted from Coptis chinensis by pH-zone-refining CCC has a strong inhibitory effect on gastric cancer cells by inducing apoptosis, affecting mitochondria and arresting the cell cycle. The research papers have demonstrated that coptisine regulates pathways such as PI3K/Akt, NF-κB, and MAPK that are essential for the progression and survival of tumour cells. Also, synergistic interactions between natural products and conventional chemotherapeutics have been gaining interest. Coptisine has been shown to enhance the anticancer effects of cisplatin and to reduce chemotherapy dosage and toxicity (Chunarkar-Patil et al., 2024). The integration of CCS with metabolomics and network pharmacology also enables the identification of multi-target anticancer compounds, thereby helping to design safer and more effective therapeutic strategies.

### 8.3 Anti-inflammatory and immunomodulatory phytochemicals

Many chronic diseases result from inflammation, including arthritis, inflammatory bowel disease, cardiovascular disorders, and autoimmune diseases. Therefore, anti-inflammatory and immunomodulatory phytochemicals have been a significant area of research on natural products. CCS technologies enable the separation of purified bioactive compounds from complex plant extracts, enabling accurate pharmacological testing (Zhang et al., 2022). With the rapid development of metabolomics, transcriptomics and network pharmacology, recent studies have begun to combine these three methods to explore the mechanisms of herbal medicines. In fact, the traditional Chinese medicine preparation Kangfuxiaoyan Suppository has been extensively studied using metabolomics analyses based on LC-MS and systems biology. Multiple active phytochemicals were identified that regulate inflammatory mediators, including tumor necrosis factor alpha (TNF-α), interleukin 6 (IL-6), interleukin 1 beta (IL-1β), and NF-κB signaling pathways (Moudgil and Venkatesha, 2022). Omics analyses showed that these compounds have synergistic activity on the immune regulation, oxidative stress, and tissue repair pathways. These results are consistent with the theories of multi-target and multi-component action of herbal medicines. CCS-assisted purification, in combination with omics technologies, therefore offers a holistic approach to elucidating the molecular basis of anti-inflammatory phytochemicals and validating their therapeutic potential (Moudgil and Venkatesha, 2022).

### 8.4 Antimicrobial and antiviral natural products

The rise of antimicrobial resistance has spurred the demand for new antimicrobial and antiviral agents from natural sources. Marine organisms, medicinal plants, fungi and microorganisms are rich in chemical diversity and valuable sources of bioactive metabolites. CCS techniques can be used to separate them while efficiently maintaining their biological activity (Stan et al., 2021). BMN combined with CCS-guided purification is a useful tool for identifying antibacterial compounds with high potency against *Staphylococcus aureus* in the brown seaweed Fucus vesiculosus. These chemicals damage bacterial cell walls and interfere with bacterial metabolism. Likewise, many CCS-isolated flavonoids, terpenoids, and alkaloids have been shown to exhibit antiviral properties against influenza viruses, hepatitis viruses, herpes simplex virus, and emerging viral diseases (Woo et al., 2023). With the aid of modern approaches integrating CCS, metabolomics, molecular docking, and network pharmacology, anti-viral compounds and molecular targets can be identified rapidly. These combined approaches can speed the development of new anti-infective drugs to meet the global threat of “super bugs (Song, 2024).

### 8.5 Neuroprotective and cardioprotective compounds

Neurodegenerative and cardiovascular diseases are complex disorders that include dysfunction of signaling pathways, inflammation, oxidative stress, and mitochondrial dysfunction. Natural products have become a widely discussed area of therapeutic interest because they act on multiple biological targets. The CCS technologies enable the high-purity isolation of specialized metabolites that need detailed pharmacological studies. A variety of marine-derived compounds, such as eckmaxol from Ecklonia maxima, have exhibited significant neuroprotective effects through antioxidant activity, antiapoptotic activity, and modulation of neuroinflammatory pathways (Mohd Sairazi and Sirajudeen, 2020; Zhang et al., 2024). Likewise, berberine, an alkaloid, has shown potential therapeutic effects in Alzheimer’s disease by modulating the PI3K/Akt, MAPK and AMPK signaling pathways, which are involved in cognitive function and neuronal survival. In cardiovascular studies, flavonoids, phenolic acids and terpenoids extracted with the CCS show cardioprotective properties. Integrating CCS with transcriptomics, proteomics, and metabolomics allows a systems-level understanding of these mechanisms and the development of new therapeutic approaches for chronic neurological and cardiovascular diseases (Angeloni et al., 2023).

### 8.6 Multi-target and synergistic therapeutic approaches

Often, the pharmaceutical model of developing a single drug targeting a single molecular target is insufficient for complex diseases with multiple interconnected biological pathways. Many natural products and herbal medicines often work by having several constituents work together. Network pharmacology has become a powerful approach to studying these multi-target mechanisms by building a network of herb-compound-target-disease interactions (Wang B. et al., 2024). CCS technologies are of great value for isolating pure compounds and enriched fractions for mechanistic evaluation. For example, thermal modification of the composition of some tirucallane-type triterpenoids and other bioactive metabolites in the frankincense was shown to drastically affect its anti-inflammatory (A) and intestinal barrier-regulating (B) activities. In fact, when investigating Paeoniae Radix Alba, researchers found that several monoterpenes were used to regulate the balance between inflammatory mediators and cartilage degradation pathways linked to the development of OA (Luo et al., 2019; Wei et al., 2023). Dragon’s Blood preparations also showed complex ADME-mediated interactions that led to therapeutic effects in colitis, as revealed by systems pharmacology analyses. The combination of CCS, metabolomics, transcriptomics and network pharmacology can comprehensively describe the synergy among phytochemicals and develop multi-target therapy strategies that are more effective for complex human diseases (Badria et al., 2025).

Network pharmacology is essential for computational approaches to understand herbal medicine and multi-target processes of natural products. Most computational projection-based network pharmacology studies are not validated experimentally. Public databases may contain errors, outdated information, and conflicting data, which may influence target prediction and route analysis. Biological networks usually lack dynamic complexity, such as disease progression, tissue-specific responses, and pharmacokinetic variability. This calls for validation of computational results by *in vitro*, *in vivo*, and clinical trials. Further studies combining network pharmacology, multi-omics data, molecular docking, molecular dynamics simulations and experimental validation can improve the prediction accuracy and translational relevance (Noor et al., 2022).

## 9 Quality control and standardization of medicinal plants

### 9.1 Authentication and chemical fingerprinting

Medicinal plant authentication and chemical fingerprinting are vital parts of the quality control of medicinal plants, ensuring the safety, purity and identity of medicinal products. Misidentification and adulteration remain significant problems in herbal medicine due to the similarity in appearance among plant species and economic pressures to replace plants. Modern authentication methods involve microscopic and macroscopic examinations, as well as advanced analytical techniques such as HPLC, UHPLC-MS/MS, GC-MS, NMR, and DNA Barcoding. Chemical fingerprinting can produce a full profile of typical metabolites, which can help to identify authentic materials and adulterants. This is complemented by CCS, which helps identify highly pure reference compounds as analytical markers. Fingerprints are high-quality markers that ensure compliance, standardization and consistency in pharmaceuticals (Brent and Pauli, 2005; Berthod and Sydow, 2023; Goodacre et al., 2023; Mohammed, 2026).

### 9.2 Reproducibility and batch-to-batch consistency

Reproducibility and batch-to-batch consistency are important criteria for assuring the therapeutic reliability of medicinal plant products. Phytochemical composition can vary significantly due to climate, soil type, harvest time, location, farming methods and processing. Such variation can impact efficacy, safety, and pharmacology. Modern quality assurance programs rely on metabolomic profiling, chromatographic fingerprinting and chemometric analysis to monitor the consistency of production batches. PCA and OPLS-DA are statistical techniques that enable the detection of subtle differences in chemical composition (Archer and Kumadoh, 2023). Standardization of cultivation and processing procedures, along with analytical monitoring, ensures that essential bioactive components do not exceed desired limits. These are vital for the development of reproducible herbal medicines, which can be used clinically and subjected to regulatory approval.

### 9.3 Quantitative metabolomics and marker compounds

Quantitative metabolomics is an approach aimed at identifying and quantifying metabolite concentrations in medicinal plant extracts, a crucial step for quality control and standardization. Quantitative methods differ from qualitative profiling in that they determine the absolute or relative amounts of specific phytochemicals associated with therapeutic activity or toxicity. Marker compound quantification is possible using sensitive, precise analytical platforms such as LC-MS/MS, GC-MS, or NMR spectroscopy (López-Yerena et al., 2021). Regulatory agencies often require the determination of one or more marker constituent(s) to ensure consistency and authenticity of the product. If reference standards are not available, a comprehensive chemical fingerprinting can be used. Quantitative metabolomics also allows evaluation of the effects of environmental conditions, processing and storage on the contents of phytochemicals, thereby ensuring the quality, safety and reproducible pharmacological activity of the product (Assouguem et al., 2025).

### 9.4 CCS in herbal quality assessment

Because of its high recovery, selectivity and absence of solid stationary phases, CCS has emerged as an important analytical and preparative method in the quality assessment of herbs. Efficient separation of complex phytochemical mixtures into chemically enriched fractions can be achieved using techniques such as HSCCC and CPC (Mi et al., 2023). This fractionation helps reduce matrix complexity and suppress signals during subsequent LC-MS, GC-MS, or NMR analysis. CCS also provides highly purified marker compounds for method validation and quantitative analysis. Moreover, the technique preserves bioactive molecules without their irreversible adsorption and degradation. Therefore, CCS is more accurate for analysis and helps to standardize and verify the quality assurance programmes of medicinal plants (Debnath et al., 2025).

### 9.5 Regulatory perspectives and ConPhyMP guidelines

The standardization of regulations is an important aspect for ensuring the quality, safety, efficacy and reproducibility of medicinal plant studies and products. To overcome inconsistencies in the reporting of phytochemicals, the Consensus-based Reporting Guidelines for the Phytochemical Characterisation of Medicinal Plant Extracts (ConPhyMP) were developed as an internationally accepted standard. ConPhyMP offers an exhaustive checklist for plant authentication, collection information, extraction process, phytochemical characterization, analytical techniques and quality assurance (Table 15) (Heinrich et al., 2022). The guidelines promote transparency and reproducibility of phytopharmacological studies, making the experimental methodology explicit and adequate. The use of ConPhyMP standards increases the comparability of studies, enables regulatory assessment and enhances the scientific credibility of herbal medicines. In the end, the guidelines facilitate the translation of medicinal plant research into clinically relevant and commercially viable phytopharmaceuticals (Berthod and Hassoun, 2006).

**TABLE 15:** Classification of medicinal plant extracts according to ConPhyMP guidelines, including definitions and recommended reporting requirements for phytochemical characterization and quality assessment.

Extract type	Definition	Key reporting requirements	References
Type A	Botanical drugs and extracts covered by a monograph in national or regional pharmacopoeias are used as active ingredients in regulated medicines.	Strict compliance with pharmacopoeial standards; chemical fingerprinting overlay with official reference standards; quantification of defined marker compounds.	Heinrich and Jalil (2023)
Type B	Botanicals used commercially internationally lack formal pharmacopoeial monographs (e.g., for commercial herbal preparations and medicinal foods).	Rigorous chemical characterization; multi-parameter chromatographic fingerprinting; absolute quantification of marker compounds.	Muyumba et al. (2021)
Type C	Species or extracts derived from plants not widely used or commercially traded (e.g., novel ethnobotanicals).	Comprehensive structural elucidation of active principles; transparent extraction reporting; *de novo* metabolomic profiling.	Karalija et al. (2025)

### 9.6 Good manufacturing practice in phytopharmaceuticals

Good Manufacturing Practice (GMP) is followed in herbal processing to ensure consistency of processes from seed cultivation to clinical formulation. Pharmaceutical-grade stainless steel is used to construct CPC systems that can perform continuous, GMP-compliant liquid-liquid processing at scale. Highly repeatable extraction efficiencies are essential for global regulatory compliance and are achieved with a standardized biphasic matrix (Table 16) (Mukherjee, 2002).

**TABLE 16:** Critical comparison of CCS, AI, omics technologies, and network pharmacology, highlighting their principal strengths, current limitations, and future research opportunities in improve the efficiency of natural product discovery and development.

Technology	Major strengths	Current limitations	Future opportunities	References
CCS	No solid stationary phase, high recovery, minimal irreversible adsorption	Solvent system optimization, longer run times, lower throughput, costly instrumentation	AI-assisted solvent selection, automation, scalable industrial platforms	Raheem et al. (2026)
AI	Rapid prediction, method optimization, pattern recognition	Data quality dependence, algorithm bias, poor interpretability, overfitting	Explainable AI, standardized datasets, hybrid experimental-AI workflows	Rudin (2019)
Omics	Systems-level biological understanding	High cost, complex data integration, reproducibility issues	Integrated multi-omics, cloud computing, AI-driven analysis	Arıkan and Muth (2023)
Network pharmacology	Multi-target mechanism prediction	Database bias, limited experimental validation, static models	Integration with omics, molecular dynamics, and clinical validation	Thombre et al. (2026)

## 10 Advanced computational and data-driven approaches

### 10.1 Distinction between AI and other computational approaches

Computational approaches used in CCS-based natural-product research encompass several methodologically distinct categories and should not be collectively described as artificial intelligence (AI). In this review, AI specifically refers to computational methods that learn patterns or predictive relationships from data, particularly machine learning (ML) and deep learning (DL). Other approaches, including chemometrics, quantum-chemical modelling, molecular networking, network pharmacology, big-data analytics, and cloud computing, serve different analytical or infrastructural functions (Figure 7) (Karamti et al., 2025).

**FIGURE 7:**
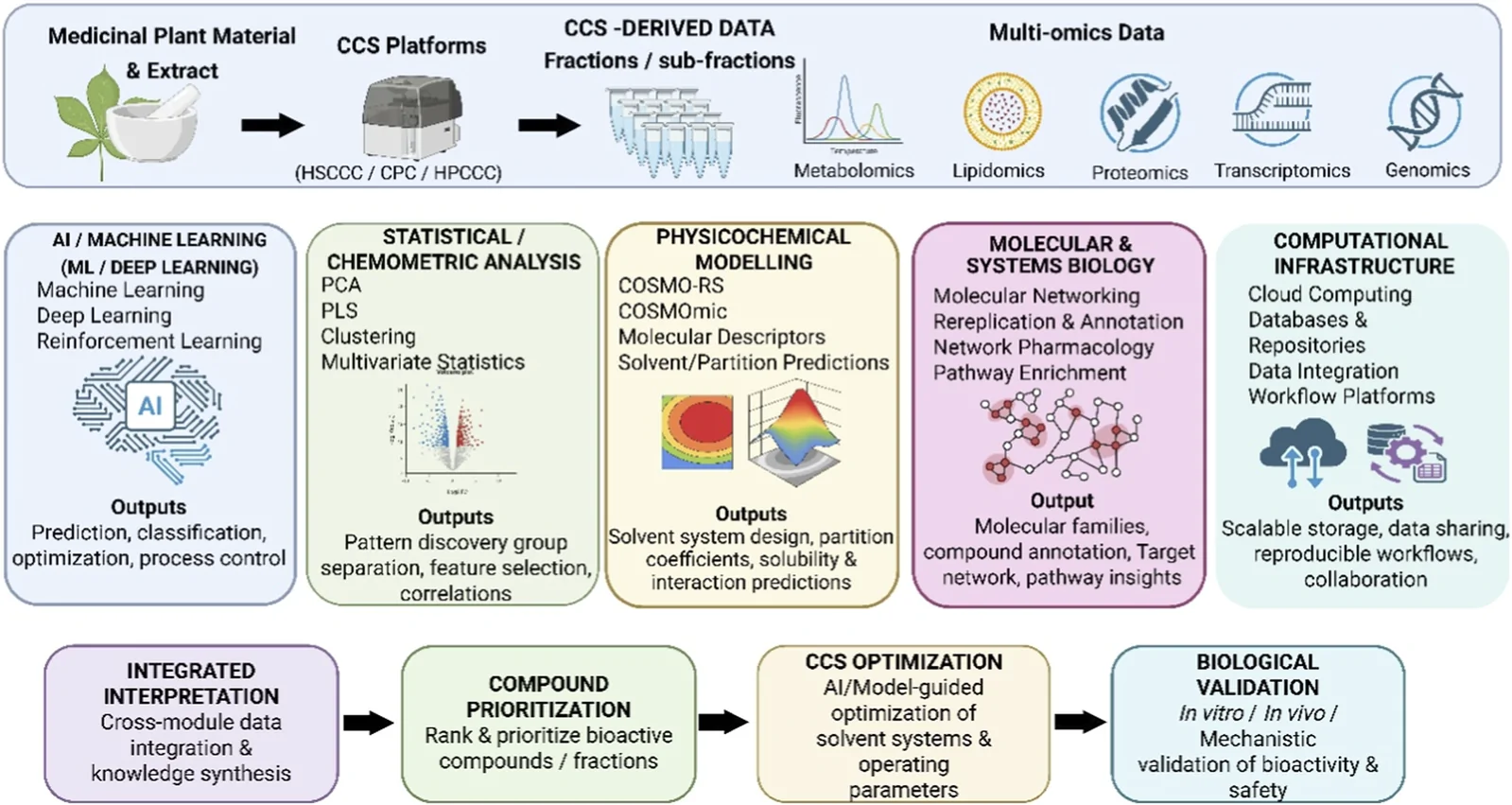
AI-powered natural product discovery platform integrating CCS, multi-omics technologies, ML, deep learning, molecular networking, chemometrics, and network pharmacology.

Machine learning and deep learning constitute the principal AI-based approaches considered in this review. ML models can learn relationships between molecular descriptors, partition coefficients, chromatographic parameters, spectral features, or historical separation datasets and predicted outcomes. Deep learning represents a subset of ML based on multilayer neural network architectures and may be useful for complex nonlinear relationships, spectral interpretation, image analysis, and automated process optimisation. Properly curated training datasets are required for these models, and they should preferably be assessed using independent test sets, external validation and suitable metrics for performance (Zhang et al., 2025).

Chemometrics is a broader analytical discipline involving the application of multivariate statistics, pattern recognition, dimensionality reduction, classification, regression, and data preprocessing to chemical measurements. Chemometric analysis therefore should not automatically be labelled as AI. Principal component analysis (PCA), hierarchical clustering, partial least-squares regression (PLS), and related conventional multivariate methods may be entirely statistical, whereas ML-based classification or prediction represents an AI-related component of chemometric workflows.

COSMO-RS and related approaches such as COSMOmic represent computational physicochemical modelling rather than AI. COSMO-RS combines quantum-chemical calculations with statistical thermodynamics to estimate molecular interactions, solvation behaviour, partitioning, and related properties. In CCS, such approaches can support rational solvent-system selection and reduce empirical solvent screening. Their predictions are governed by physicochemical models rather than data-driven learning (Table 17) (Santiago et al., 2023).

**TABLE 17:** Classification of AI and related computational approaches used in CCS-based natural-product research.

Computational category	Examples	Principal input	Typical output	Validation requirements	Major limitations	References
Machine learning (AI)	Random forest, SVM, regression, neural networks	CCS parameters, molecular descriptors, K-values, spectral/chemical features	Prediction, classification, optimization	Training/test separation, cross-validation, external validation, appropriate performance metrics	Small datasets, overfitting, poor generalizability	Wu et al. (2025)
Deep learning (AI)	Deep neural networks, CNNs, advanced neural architectures	Large spectral, image, chromatographic or multidimensional datasets	Complex prediction, classification, automated feature recognition	Independent datasets, external validation, robustness and explainability assessment	High data requirements, computational complexity, limited interpretability	Lun et al. (2025)
Chemometrics	PCA, PLS, clustering, multivariate analysis	Chromatographic, spectroscopic and metabolomic data	Patterns, clusters, classification, regression	Appropriate preprocessing, statistical validation, independent verification where applicable	Sensitive to preprocessing and dataset structure; not inherently AI	Picone (2026)
COSMO-RS/COSMOmic	Quantum-chemical/statistical thermodynamic modelling	Molecular structures and solvent properties	Solubility, partitioning and solvent-system predictions	Comparison with experimental partition/solubility data	Model assumptions and parameterization; not data-learning AI	Cordova et al. (2024)
Molecular networking	GNPS/MS/MS spectral networking	MS/MS spectra and spectral similarity	Molecular families, dereplication, annotation hypotheses	Spectral-library matching, orthogonal structural confirmation	Spectral similarity does not prove molecular identity	Morehouse et al. (2023)
Network pharmacology	Compound–target–pathway networks	Compound information, predicted/known targets, pathway databases	Mechanistic hypotheses and interaction networks	Experimental target/pathway validation	Database bias, target-prediction uncertainty, network incompleteness	Panossian (2025)
Big-data analytics	Large-scale data integration and mining	Multi-source chemical/biological datasets	Associations, trends, predictive patterns	Statistical validation, reproducibility, independent datasets	Heterogeneous formats, batch effects, missing data	Jiang (2025)
Cloud/digital infrastructure	Cloud databases, shared platforms, computational workflows	Large analytical datasets	Storage, sharing, workflow execution and collaboration	Data integrity, reproducibility, interoperability and security	Infrastructure dependency; not an analytical method	Vignolo et al. (2020)

Molecular networking is a computational framework for organising MS/MS spectra according to spectral similarity. It facilitates molecular-family visualisation, dereplication, annotation, and prioritisation of structurally related metabolites. Although machine-learning algorithms may be incorporated into some molecular-networking workflows, molecular networking itself should not be considered synonymous with AI.

Network pharmacology and systems biology are mechanism-orientated computational frameworks that integrate information concerning compounds, molecular targets, biological pathways, and disease-associated processes. Their principal output is a compound–target–pathway or related interaction network that generates hypotheses concerning potential mechanisms of action. These predictions require experimental biological validation and may be affected by database completeness and prediction bias (Qin et al., 2022).

‘Big-data analytics’ refers to the processing, integration, and statistical analysis of large and heterogeneous datasets generated from metabolomics, genomics, proteomics, ethnobotanical databases, and other sources. Big data analysis can be done with AI or traditional statistics but the term is not a specific AI algorithm.

Cloud computing and digital platforms provide the computational infrastructure that supports the analytical methods. Cloud environments offer data storage, data sharing, workflow execution, computational processing and collaborative analysis. Cloud-enabled molecular-networking environments provide for sharing of MS data and computational analysis. However, the cloud infrastructure is not, in and of itself, an AI method (Hassan et al., 2022).

## 11 Industrial translation and future perspectives

### 11.1 Industrial scale-up of CCS technologies

Predictable liquid-liquid partitioning is useful for the scale-up of CCS technologies. Conventional column chromatography is limited by pressure and can produce irreversible adsorption. CCS systems do not have these pressure related limitation and irreversible adsorption problems. To scale up, the number of rotors and/or coils is increased, while keeping the partition behavior and separation efficiency constant (Tallon and Moreno, 2014). This linear scalability enables high-purity production of phytochemicals at an industrial scale, facilitating pharmaceutical and nutraceutical production, as well as large-scale natural product purification, with improved cost-effectiveness and process reproducibility.

### 11.2 Sustainable and eco-friendly separation strategies

One of today’s major concerns in the production of phytopharmaceuticals is sustainability. CCS technologies can follow the green chemistry principles by minimizing solvent usage and eliminating solid stationary phases. Environmental impact is reduced by using environmentally friendly solvent systems, such as DESs, Natural Deep Eutectic Solvents (NADESs), and Green Arizona solvent systems (Gupta, 2025). Solvents are also being recycled continuously, resulting in additional reductions of waste generation and operational costs. Scalable natural product extraction methods are environmentally friendly and meet global environmental and regulatory standards.

### 11.3 Translational potential in drug discovery pipelines

The combination of CCS with omics technologies and AI greatly improves the drug discovery process. CCS offers highly pure compounds with high recovery and molecular networking, and metabolomics accelerates the identification of bioactive molecules. The large compound libraries generated by CCS-based fractionation can be screened on automated platforms. This streamlined process helps identify leads quickly, shortens development time and increases the likelihood that natural products will be used in clinically relevant therapeutic candidates for pharmaceutical development (Sabry et al., 2025).

### 11.4 Personalized phytomedicine and precision pharmacology

Phytomedicine is a tailored approach of using herbal medicines that is based on the genetic, metabolic and physiological differences of each person. Omics technologies, such as genomics, metabolomics, and pharmacogenomics, provide insights into patients’ responses to botanical medicines. Due to the standardized extracts obtained through a GMP-compliant CCS process, there is a consistent phytochemical composition and therapeutic activity (Shinde et al., 2026). Combining systems biology and network pharmacology enables precision phytotherapeutic approaches that simultaneously target multiple pathways, thereby enhancing treatment efficacy and reducing side effects across diverse patient populations.

### 11.5 Challenges in commercialization and regulatory approval

The commercialization of phytopharmaceuticals is challenging due to strict regulations, but interest is increasing. Regulatory agencies require comprehensive evidence of safety, efficacy, quality and batch-to-batch consistency. Botanical extracts can be highly complex, making standardization and quality assessment difficult. The guidelines, such as ConPhyMP, facilitate transparent reporting of plant authentication, extraction methods and phytochemical characterization. To meet these challenges, advanced analytical tools, quantitative metabolomics and CCS-assisted purification aid in the regulatory acceptance and market approval of herbal medicinal products (Table 18) (Prajapati et al., 2023).

**TABLE 18:** Current challenges, impacts, and future opportunities in omics-integrated CCS research, highlighting emerging roles of AI, ML, automation, cloud-based multi-omics platforms, and standardized quality frameworks.

Current challenges	Impact	Future opportunities	References
Time-consuming solvent optimization	Reduced efficiency	AI-driven solvent prediction	Pérez-Baeza et al. (2022)
Complex omics datasets	Difficult interpretation	Advanced ML	Nascimento et al. (2024)
Metabolite annotation bottleneck	Slow compound identification	Automated molecular networking	Wang et al. (2016)
Lack of standardization	Poor reproducibility	ConPhyMP-guided workflows	Heinrich et al. (2022)
Scale-up limitations	Industrial translation barriers	Continuous CCS processing	He et al. (2024)
Data integration issues	Fragmented biological insight	Cloud-based multi-omics platforms	Subramanian et al. (2020)
Regulatory hurdles	Delayed commercialization	Standardized quality frameworks	Ekor (2014)
High analytical costs	Limited accessibility	Automation and high-throughput systems	Wolfender et al. (2015)

### 11.6 Future directions in omics-integrated CCS research

Future development for CCS research with the help of omics will be directed towards automation, AI and real-time analytical monitoring. The use of AI for solvent selection, ML for process optimisation and automated multidimensional CCS systems are anticipated to enhance separation efficiency and reproducibility.

The system will be integrated with a cloud-based platform, molecular networking tools and real-time LCMS analysis to facilitate autonomous compound identification and isolation. The innovations will aid in the discovery of new natural products, improve pharmaceutical innovation, and enable new applications of precision medicine and phytopharmaceuticals (Gangwal and Lavecchia, 2025).

## 12 Regulatory considerations for CCS-based botanical drug development

### 12.1 FDA botanical drug development guidance

FDA has standards for research, testing and commercialization of botanical medicines. Unlike conventional drugs which have a single active ingredient, botanical therapies are complex mixtures. The therapeutic efficacy may be provided by a combination of several components. The FDA stresses on the GMP, chemical fingerprinting, stability testing, homogeneous production, quality control and thorough botanical characterization during the product development. Chemometrics, metabolomics and CCS are modern analytical platforms that improve botanical characterization. These techniques allow reproducible extraction of bioactive components, identification of chemical markers and determination of batch to batch consistency. Therefore, these processes meet FDA botanical medicine quality, safety and efficacy requirements. Guidance for Industry Botanical Drug Development (Kaundal and Kumar, 2025).

### 12.2 EMA herbal medicinal products framework

In Europe, the Committee on Herbal Medicinal Goods (HMPC) formulates community herbal monographs, evaluation reports and quality recommendations. It is regulated by the European Medicines Agency. EMA gives importance to botanical validity, standardization of herbal preparation, manufacturing quality, safety assessment and uniformity of phytochemical composition. Current analytical approaches such as CCS in combination with metabolomics are essential to characterize marker molecules, improve chemical profiling and repeat fractionation. Traditional herbal medicines have more scientific evidence and omics-based analytical methods identify quality signals.

### 12.3 Comparative regulatory perspectives and future harmonization

ConPhyMP, FDA Botanical Drug Development Guidance and EMA Herbal Medicinal Products all seek to improve the science of botanical medicine, but address different regulatory review issues. While the FDA focus is on manufacturing uniformity, clinical development and product quality of botanical medicines, the ConPhyMP focus is on phytochemical characterization and open reporting guidelines for medicinal plant extracts. European herbal medicines, however, must meet EMA requirements for quality, safety, efficacy and traditional use (Table 19). The future convergence of these regulatory strategies in CCS, metabolomics, AI and systems biology may result in harmonized phytochemical characterization, reproducible quality assessment and globally accepted botanical product analytical workflows.

**TABLE 19:** Comparison of major regulatory frameworks relevant to botanical drug development and the potential contribution of CCS and omics technologies.

Regulatory framework	Primary objective	Major analytical requirements	Role of CCS and omics	Guideline/reference
ConPhyMP	Standardized phytochemical characterization	Chemical profiling, marker compounds, extract characterization	High-recovery fractionation, metabolomics-assisted characterization	Springer Nature (2022)
FDA Botanical Drug Development Guidance	Development and regulatory approval of botanical drugs	Manufacturing consistency, quality control, GMP, stability, chemical fingerprinting	CCS for reproducible isolation; metabolomics for batch consistency and marker identification	Center for Drug Evaluation and Research (2020)
EMA Herbal Medicinal Products Framework	Regulation of herbal medicinal products in the EU	Botanical authentication, standardization, safety, efficacy, quality documentation	CCS-assisted phytochemical profiling and quality marker identification	European Medicines Agency (2023)

### 12.4 Regulatory gaps and translational barriers for CCS–omics-derived botanical products

Although CCS–omics workflows have the potential to improve the characterization and standardization of botanical products, several regulatory and translational gaps still exist. The botanical identity and authentication of the starting material should be established, and the consistency of the starting material ensured by appropriate taxonomic identification, source documentation, and chemical characterization. This should be supplemented with chemical fingerprinting and quantitative determination of relevant marker compounds, thus allowing comparison of different batches and detection of compositional change. Variations in cultivation, geographic origin, harvesting, processing, and extraction can, however, lead to batch-to-batch variation, and therefore standardized manufacturing is especially important. CCS can aid in the isolation and characterization of marker compounds, while omics-based approaches can provide a more complete chemical fingerprint. Nevertheless, these analytical approaches need to be incorporated into validated quality-control procedures. At the manufacturing level reproducible quality during production and storage requires GMP-compliant processes, appropriate stability assessment, validated analytical methods, and defined specifications. The manuscript emphasizes the value of ConPhyMP-based reporting, chemical fingerprinting, marker-compound quantification, and GMP principles for improving the reproducibility and regulatory evaluation of botanical products (Figure 8) (Applequist and Miller, 2013).

**FIGURE 8:**
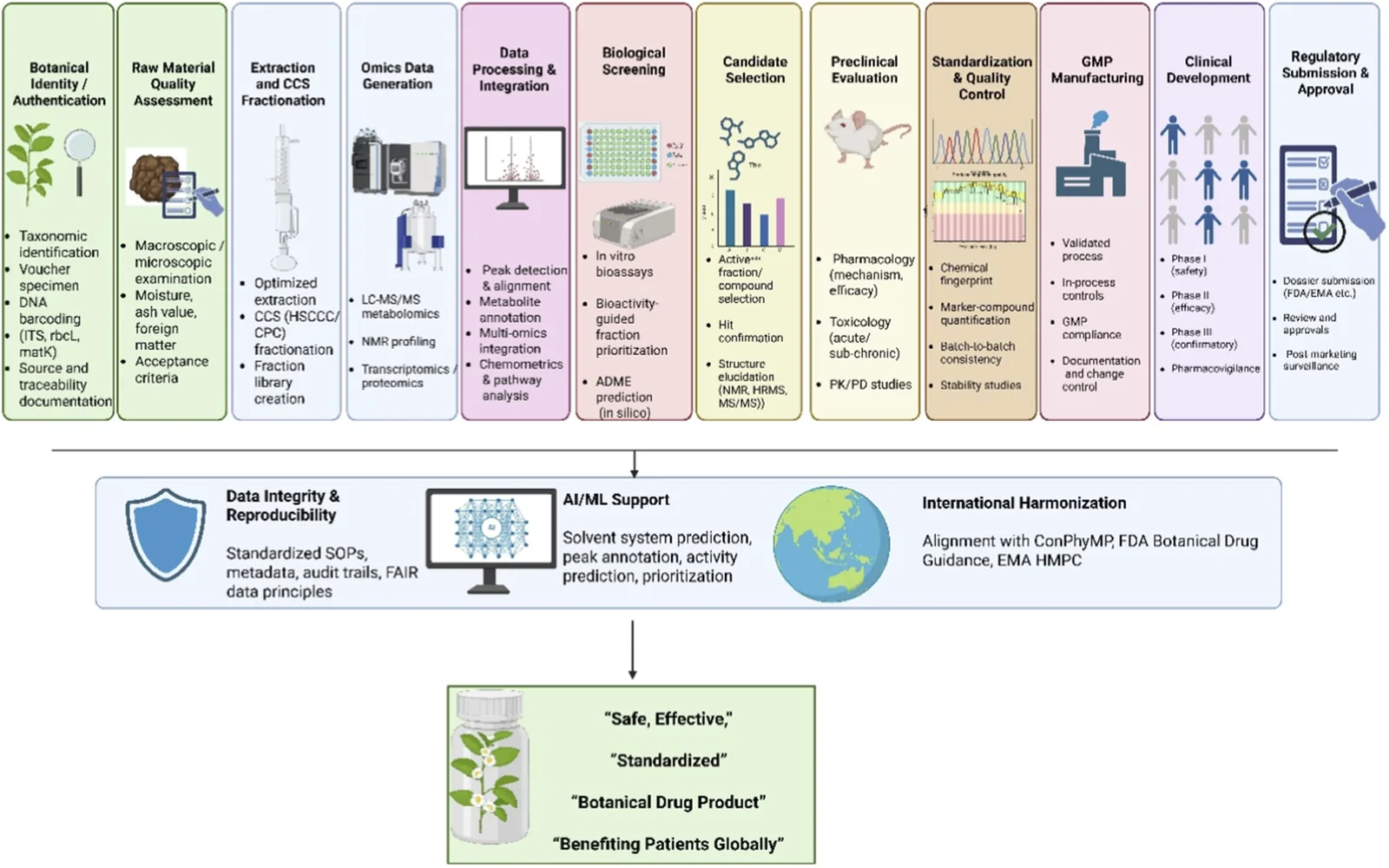
Proposed regulatory and translational workflow for CCS–omics-enabled botanical drug development.

A further translational barrier is the gap between chemical discovery and demonstrated clinical benefit. Identification of candidate metabolites through CCS–omics does not establish therapeutic efficacy, and biological activity observed in screening assays requires confirmation through appropriate pharmacological, toxicological, pharmacokinetic, and eventually clinical studies. Similarly, omics-derived biomarkers require independent validation before they can be considered reliable indicators for quality control, biological activity, or therapeutic response. AI/ML-based predictions for solvent-system selection, metabolite annotation, biomarker prioritization, or chromatographic optimization also require experimental and external validation before their use in regulated decision-making. In addition, standardized data-processing procedures, interoperable analytical platforms, transparent reporting, and appropriate quality-control procedures are necessary to address data integrity and reproducibility. The current manuscript identifies small datasets, lack of external validation, heterogeneous analytical platforms, and differences in preprocessing and statistical workflows as important barriers to reproducibility (Mahalmani et al., 2022).

Finally, international regulatory harmonization remains an important challenge for the translation of CCS–omics-derived botanical products. Differences among national and regional requirements for botanical authentication, chemical characterization, marker-compound specifications, manufacturing controls, safety assessment, and clinical evidence can complicate the development of globally acceptable products. The integration of ConPhyMP reporting principles with regulatory frameworks such as FDA Botanical Drug Development guidance and the EMA framework for herbal medicinal products could improve transparency and comparability across jurisdictions. However, there is a need for broader harmonization of analytical standards, quality specifications, omics-data reporting, validation requirements, and manufacturing practices. However, we still need to harmonize analytical standards, quality specifications, omics-data reporting, validation requirements, and manufacturing practices more broadly. Therefore, the successful translation of CCS–omics technologies into botanical drug development will require not only technological advances but also standardized quality systems, validated biomarkers and computational approaches, robust safety and clinical evidence, reproducible manufacturing, and greater alignment of international regulatory expectations (Osman et al., 2023).

## 13 Research gaps and priority areas for future investigation

Although CCS–omics integration has substantially expanded the analytical and biological investigation of medicinal plants, several important gaps remain before these approaches can be routinely translated into standardized, industrial, and clinically relevant botanical drug-development workflows. The major challenges include methodological standardization, reproducibility, independent validation, scale-up, regulatory acceptance, and clinical qualification. Future research should prioritize the following areas. In future research.

### 13.1 Lack of standardized CCS operating conditions

A major research gap is the lack of a universally standardized operating condition of fractionation based on CCS. Solvent system, phase composition, partition coefficients, flow rate, rotation speed, temperature, sample loading, and stationary-phase retention can vary greatly depending on the botanical matrix and target compounds. This variability can impact the separation efficiency, recovery, purity and reproducibility between studies. Although trial-and-error solvent optimization can be reduced by AI/ML and computational approaches such as COSMO-RS, wider experimental validation of these approaches is needed. Future studies should therefore establish standardized operating ranges, validated solvent-selection procedures, reference materials, and reporting criteria for different classes of botanical matrices (Floyd et al., 2026).

### 13.2 Limited cross-platform omics validation

The integration of metabolomics, proteomics, transcriptomics, lipidomics and other omics platforms provides a wealth of biological information, but interpretation can be confounded by differences in analytical resolution, dynamic range, data formats, normalization and pre-processing strategies. Cross-platform validation is particularly useful to confirm whether metabolite signatures and biomarkers discovered on one analytical platform are reproducible on an alternative platform or complementary analytical approach. Some major challenges are also pointed out in the manuscript, namely, heterogeneous multi-omics datasets and the need for standardized databases and interoperable workflows. Future research should therefore focus on orthogonal analytical validation and on standardized data-processing pipelines (Wörheide et al., 2021).

### 13.3 Limited multicentre reproducibility

Most CCS-omics studies remain laboratory-specific and only few reproducibility studies have been reported in independent research centers. Variability across laboratories can be introduced by differences in instrumentation, solvent preparation, sample handling, CCS operating parameters, omics platforms, reference standards and bioinformatic pipelines. Interlaboratory variability is explicitly mentioned in the manuscript as a reproducibility problem, and the use of standardized operating procedures and reference materials is suggested. Multicenter studies with common reference samples, predefined protocols, and blinded datasets are required to assess the robustness and transferability of CCS–omics workflows (Peng et al., 2022).

### 13.4 Insufficient quantitative comparison among CCS platforms

Although advantages of CCS such as high recovery and low irreversible adsorption have been demonstrated, quantitative comparisons among different CCS platforms and between CCS and conventional preparative techniques are still insufficient. Future studies should compare the different separation approaches (HSCCC, CPC, centrifugal counter-current chromatography, preparative HPLC, and others) systematically with standardized parameters (recovery, purity, resolution, processing time, solvent consumption, sample-loading capacity, cost, energy requirements, and scalability). Such quantitative benchmarking would help to establish in which specific circumstances one separation technology provides a meaningful advantage over another, rather than assuming that CCS is universally superior. The manuscript already lists solvent optimization, longer processing times, lower throughput and instrumentation costs as limitations that should be considered against the benefits of CCS (Rani et al., 2025).

### 13.5 Limited validation of AI/ML predictions

AI and ML have considerable potential for solvent-system prediction, chromatographic optimization, metabolite annotation, biomarker identification, and automated CCS operation. However, current evidence remains limited because many models are based on retrospective datasets, proof-of-concept experiments, or relatively small datasets. Independent multicenter validation, prospective experimental testing, transparent performance evaluation, and explainable AI are required before these approaches can be considered reliable analytical tools for routine applications. Future studies should establish benchmark datasets containing diverse natural-product classes and standardized performance metrics and should experimentally verify AI-generated predictions before implementation in regulated workflows (Kanapari et al., 2025).

### 13.6 Challenges in industrial scale-up

Conversion of laboratory-scale CCS processes to industrial production still presents a challenge, as changes in equipment geometry, flow dynamics, solvent handling, mass transfer, sample loading, and process control can influence separation performance at larger scales. While CPC can be applied for continuous liquid–liquid processing and GMP-compatible production, industrial application needs validated process parameters and reliable performance in extraction and fractionation. The high costs of instrumentation and the need for advanced analytical infrastructure also hinder wider industrial adoption. Thus, future work should be directed toward continuous CCS processing, process automation, real-time monitoring, scalable design of equipment, and cost-effective high-throughput workflows (Palma et al., 2020).

### 13.7 Regulatory and quality-control gaps

Regulatory translation requires standardization of botanical identity, chemical fingerprinting, quantification of marker compounds, consistency from batch to batch, stability testing, use of validated analytical methods, manufacturing under GMP and appropriate documentation of safety and efficacy. Although ConPhyMP, FDA botanical drug guidance, and EMA herbal medicinal-product frameworks provide important foundations, differences in regulatory requirements may complicate international development. The manuscript emphasizes that FDA requirements include manufacturing consistency, quality control, GMP, stability, and chemical fingerprinting, whereas EMA requirements emphasize botanical authentication, standardization, safety, efficacy, and quality documentation. Future work should therefore develop harmonized quality specifications and validated CCS–omics analytical workflows that can support regulatory submissions across different jurisdictions (Zahmanova et al., 2023).

### 13.8 Need for pharmacological and toxicological validation

Chemical identification and metabolomic association alone do not establish therapeutic efficacy or safety. The Omics assisted approaches for the identification of candidate compounds and CCS derived fractions need to be validated by the proper pharmacological studies, mechanism of action studies, pharmacokinetic evaluation, and acute and chronic toxicological evaluation. Therefore, the predicted biological activities and molecular targets from computational or network-pharmacology approaches would need experimental validation. Future studies should therefore establish a sequential validation framework linking chemical identity → biological activity → mechanism → pharmacokinetics → toxicity → therapeutic efficacy before moving promising candidates towards clinical development. The manuscript already points out the need for biological validation and analytical validation for regulatory qualification of omics-derived biomarkers (Stolaki et al., 2025).

### 13.9 Need for clinical translation

The third and final research gap is the lack of translation of CCS–omics findings into clinically validated botanical products. While the identification of bioactive metabolites, biomarkers or molecular networks is an important step, it does not equate to clinical efficacy, safety or therapeutic benefit. Valid biomarkers, standardized botanical preparations, reproducible manufacturing, adequate pharmacological and toxicological evidence, and well-designed clinical studies are required for clinical translation. Regulatory qualification of omics-derived biomarkers additionally requires analytical and biological validation before they can be used to support pharmaceutical or precision-medicine use. Therefore, future research should establish prospective, multicenter clinical-validation studies that connect CCS-derived chemical profiles and omics signatures with pharmacological response, safety outcomes, and clinically meaningful endpoints (Welsing et al., 2025).

## 14 Conclusion

Modern methods must handle chemical complexity and sample integrity to identify and standardise medicinal plant bioactive chemicals. Irreversible adsorptive losses, scalability issues, and preparative limits plague solid-support systems. Without solid matrices, CPC and HSCCC use dynamic liquid-liquid partitioning to achieve nearly full separation and high mass-loading capacity. These scalable separation methods and powerful multi-omics platforms must be integrated to develop pharmacognosy. Metabolic fingerprinting and computational molecular networking (GNPS) eliminate bioassay-guided fractionation’s empirical blind spot, enabling rational *in silico* targeting of bioactive chemotypes before physical isolation. “Solvent-system design can be supported by complementary data-driven and mechanistic approaches, including machine-learning models trained on experimental separation data and COSMO-RS-based physicochemical simulations.” A single pipeline of omics-integrated CCS follows ConPhyMP and network pharmacology systems biology concepts to improve analytical resolution. This synergistic strategy advances ethnopharmacology and high-throughput botanical drug discovery.

CCS, Multi-Omics and AI Might Revolutionize Natural Product and Phytopharmaceutical Identification These advances in CCS-assisted metabolomics, genomes, transcriptomics, proteomics and lipidomics have identified, purified and characterized bioactive phytochemicals and their mode of actions. AI-driven predictive modeling and bioinformatics for solvent-system optimization, metabolite identification, biomarker creation and data interpretation are facilitating more efficient and reproducible CCS-based methods. Despite advances the field has many questions.” To clinically validate biomarkers from omics data we need regulatory structures, standardized pipelines for data integration from different omics sources, consistent analytical and reporting protocols, large scale validation studies in multiple centers and rigorous experimental validation of AI predictions. Automated analytics improve industrial scalability, affordability and interoperability. CCS-based fractionation of botanical extracts, multi-omics profiling including comprehensive omics characterization to reveal biomarkers and bioactive compounds, AI-assisted prioritization and predictive modeling, experimental validation via pharmacological, toxicological and mechanistic studies, regulatory standardization and quality assurance may be part of the phytopharmaceutical development.
